# LILRB4 suppresses immunity in solid tumors and is a potential target for immunotherapy

**DOI:** 10.1084/jem.20201811

**Published:** 2021-05-11

**Authors:** Naveen Sharma, Oluwatomisin T. Atolagbe, Zhongqi Ge, James P. Allison

**Affiliations:** 1 Department of Immunology, The University of Texas MD Anderson Cancer Center, Houston, TX; 2 Immunotherapy Platform, The University of Texas MD Anderson Cancer Center, Houston, TX; 3 Parker Institute for Cancer Immunotherapy, The University of Texas MD Anderson Cancer Center, Houston, TX

## Abstract

Immune receptors expressed on TAMs are intriguing targets for tumor immunotherapy. In this study, we found inhibitory receptor LILRB4 on a variety of intratumoral immune cell types in murine tumor models and human cancers, most prominently on TAMs. LILRB4, known as gp49B in mice, is a LILRB family receptor. Human and murine LILRB4 have two extracellular domains but differ in the number of intracellular ITIMs (three versus two). We observed a high correlation in LILRB4 expression with other immune inhibitory receptors. After tumor challenge, LILRB4^−/−^ mice and mice treated with anti-LILRB4 antibody showed reduced tumor burden and increased survival. LILRB4^−/−^ genotype or LILRB4 blockade increased tumor immune infiltrates and the effector (Teff) to regulatory (Treg) T cell ratio and modulated phenotypes of TAMs toward less suppressive, CD4^+^ T cells to Th1 effector, and CD8^+^ T cells to less exhausted. These findings reveal that LILRB4 strongly suppresses tumor immunity in TME and that alleviating that suppression provides antitumor efficacy.

## Teaser

LILRB4 strongly suppresses tumor immunity, and LILRB4 blockade alleviates that suppression to provide antitumor efficacy. The authors show that LILRB4 is expressed largely on TAMs and suppresses antitumor immunity. Genetic deletion of LILRB4 or anti-LILRB4 antibody treatment modulates T cells to an effector phenotype and TAMs to a less immunosuppressive phenotype and increases survival against tumor challenge.

## Introduction

Suppression of immune cell function by engagement of coinhibitory receptors allows cancer to evade the host immune system. One arm of cancer immunotherapy involves administration of antibodies against these receptors to block their interactions with their ligands and prevent this suppression. Anti–CTLA-4 (ipilimumab) was the first inhibitory receptor blockade antibody developed and approved for tumor immunotherapy by the Food and Drug Administration due to its efficacy against metastatic melanoma (Hodi et al., 2010; Leach et al., 1996). Ipilimumab has since been shown to provide a lasting antitumor effect. Antagonistic antibodies against other inhibitory receptors and ligands, notably the PD-1/PD-L1 axis, have subsequently been demonstrated effective in tumor immunotherapy, resulting in Food and Drug Administration approval (Iwai et al., 2002; Brahmer et al., 2012; Topalian et al., 2012a, 2012b; Powles et al., 2014). Current immunotherapy drugs are effective in treating some cancer patients with certain tumor types but ineffective for many others, especially so-called cold cancers, which are characterized by a lack of T cell infiltration (Bonaventura et al., 2019). Because nonresponsiveness or resistance against many of these drugs is caused by the up-regulation of other immune inhibitory mechanisms, there is an ongoing effort to implement new combination therapies and find new immunotherapy targets (Sharma and Allison, 2015; Ribas and Wolchok, 2018).

The tumor microenvironment (TME) consists of a variety of cells, which includes many immune cells that form a complex interaction network with T cells, including immunosuppressive tumor-associated macrophages (TAMs), which constitute a large fraction of the TME (Georgoudaki et al., 2016; Yang and Zhang, 2017; Peranzoni et al., 2018). There are vast numbers of inhibitory receptors expressed by TAMs, T cells, and other cell types that have not been studied in great detail in the tumor context (Colonna et al., 2000; Crawford and Wherry, 2009; Munitz, 2010; Pentcheva-Hoang et al., 2009). Identifying and targeting relevant immune receptors provides a promising avenue to turn the tide in favor of antitumor immunity.

LILRB4 belongs to the leukocyte Ig-like receptor superfamily, which comprises type I transmembrane glycoproteins with extracellular Ig-like domains and two intracellular ITIMs (immunoreceptor tyrosine-based inhibitory motifs; Colonna et al., 2000). Integrin α_V_β_3_ and apolipoprotein E have been suggested as ligands of LILRB4 (Castells et al., 2001; Deng et al., 2018). LILRB4 is expressed on a wide variety of immune cell types, including dendritic cells (DCs), monocytes, macrophages, mast cells, B cells, natural killer (NK) cells, T cells, and osteoclasts (Cella et al., 1997; Castells et al., 2001; Gu et al., 2003; Kasai et al., 2008; Fukao et al., 2014). LILRB4-deficient T cells have been shown to have increased IFN-γ production and cytotoxicity in an acute viral infection model (Gu et al., 2003). Human LILRB4 has two extracellular Ig domains, a transmembrane domain, and three ITIMs. LILRB4 is shown to be expressed highly in leukemia cells and has been shown to suppress T cell activity (Deng et al., 2018). Soluble LILRB4 has been found in the serum of pancreatic carcinoma, colorectal carcinoma, and melanoma patients, and T cell responses are increased in vitro upon treatment with anti-LILRB4 antibody (Suciu-Foca et al., 2007). Also, casein kinase 2–regulated LILRB4 expression on regulatory T cells (Treg cells) has been shown to exhibit an immune-regulatory mechanism (Ulges et al., 2015).

In this study, we examined the expression of LILRB4 on various cell populations within the TME using mass cytometry, flow cytometry, single-cell RNA sequencing (scRNA-seq), and RNA NanoString analysis. We investigated the effect of LILRB4 on antitumor immunity in various murine tumor models by using antibody against this receptor and the LILRB4^−/−^ mouse model.

## Results

### High expression of LILRB4 in murine and human tumors

**Figure 1.**
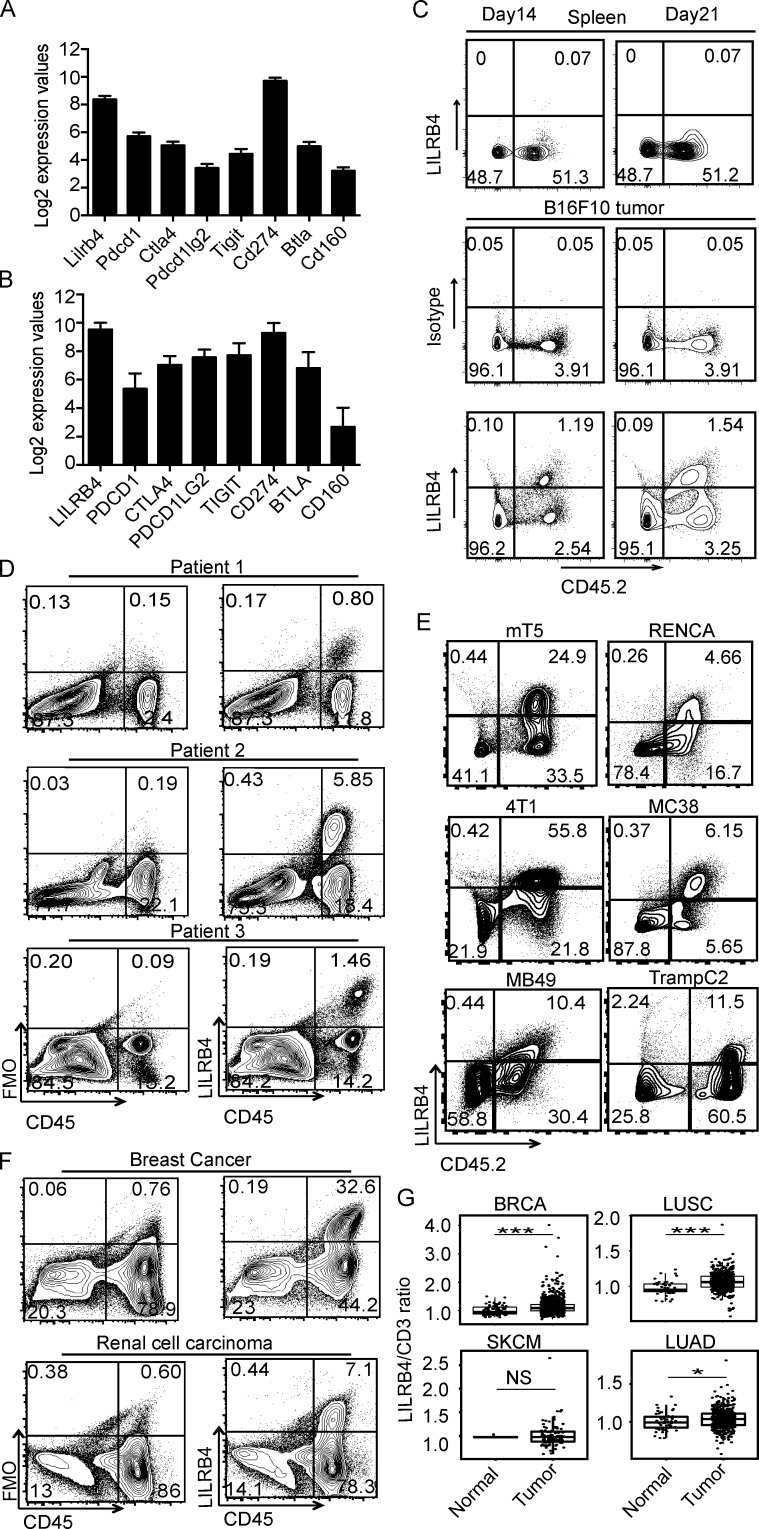
**LILRB4 is expressed on CD45^+^ cells in various murine tumor models and cancer patients. (A)** NanoString RNA analysis of tumors from mice challenged with B16/F10 cells. Error bars represent the mean ± SEM. **(B)** NanoString analysis of RNA isolated from tumors of four baseline melanoma patients. Bar plot illustrates the log2 RNA expression values. Error bars represent the mean ± SEM. **(C)** Flow cytometry analysis of tumor-infiltrating and splenic cells from mice challenged with B16/F10 cells. **(D)** Flow cytometry analysis of tumor samples taken from melanoma patients. **(E)** Flow cytometry analysis of tumors taken from mice challenged with indicated tumor cells. **(F)** Flow cytometry analysis of tumor samples from patients with indicated cancers. **(G)** Comparison of *LILRB4* to* CD3ε* mRNA ratios in human tumor samples compared with healthy tissues (TCGA database). BRCA, breast cancer; LUSC, lung squamous cell carcinoma; LUAD, lung adenocarcinoma; SKCM, skin cutaneous melanoma. Data are representative of two or three experiments with four to six mice in each experiment. *, P < 0.05; ***, P < 0.001 (Mantel–Cox test). FMO: Fluorescence Minus One.

We analyzed the expression of a number of inhibitory receptors in the mouse melanoma model B16/F10 by NanoString RNA expression analysis. Tumors were isolated from mice challenged with B16/F10 tumor cells, digested, and total RNA was isolated for expression analysis using nCounter mouse immunology panel, which had 549 genes for analysis and 14 internal reference genes. Among receptors known to have ITIM-containing domains and/or known to be inhibitory in function, we identified *Lilrb4* and *Cd274 *(*PDL1*) to be the most highly expressed in the TME (Fig. 1 A). *Lilrb4* RNA was expressed at an even higher level than *Pdcd1*, a known inhibitory receptor molecule, which is known to have a prominent role in tumor immunity. Most immune inhibitory receptors are up-regulated by chronic immune stimuli in tumors, suggesting that LILRB4 has an immunosuppressive role in the TME. We also analyzed the gene expression of this receptor in tumors from melanoma patients. Our NanoString gene expression data showed relatively high *LILRB4* expression in human melanoma in comparison to other inhibitory receptors (Fig. 1 B). We went on to analyze LILRB4 protein expression by flow cytometry on tumor-infiltrating immune cells in B16/F10 tumor implanted mice and found that LILRB4 was expressed on CD45^+^ cells, whereas its expression was not detected in spleen (Fig. 1 C). We examined the expression of LILRB4 on tumor-infiltrating immune cells at different stages of tumor development and observed that LILRB4 expression increased on CD45^+^ cells as B16/F10 tumor progressed (day 14 tumor versus day 21 tumor; Fig. 1 C). Similarly, we also found LILRB4 protein expression on tumor-infiltrating CD45^+^ cells in melanoma patients (Fig. 1 D). We next explored the expression of LILRB4 on tumor-infiltrating immune cells from a variety of murine and human tumors by flow cytometry; it was highly expressed on CD45^+^ cells in most murine tumor models and cancer patients (Fig. 1, E and F).

To understand the extent of expression of LILRB4 in different tumor types, we analyzed RNA expression data from The Cancer Genome Atlas (TCGA) database. Breast cancer, lung squamous cell carcinoma, and lung adenocarcinoma showed a significantly higher *LILRB4*/*CD3ε* mRNA expression ratio in tumor samples than normal tissue samples (Fig. 1 G). Skin cutaneous melanoma and bladder cancer (data not shown), although not significant, showed a trend of higher LILRB4 compared with normal tissue. However, the availability of few normal tissue samples in these tumor types compared with patient tumor samples made it difficult to derive any conclusion.

### LILRB4 is expressed on various tumor-infiltrating immune cell types

**Figure S1.**
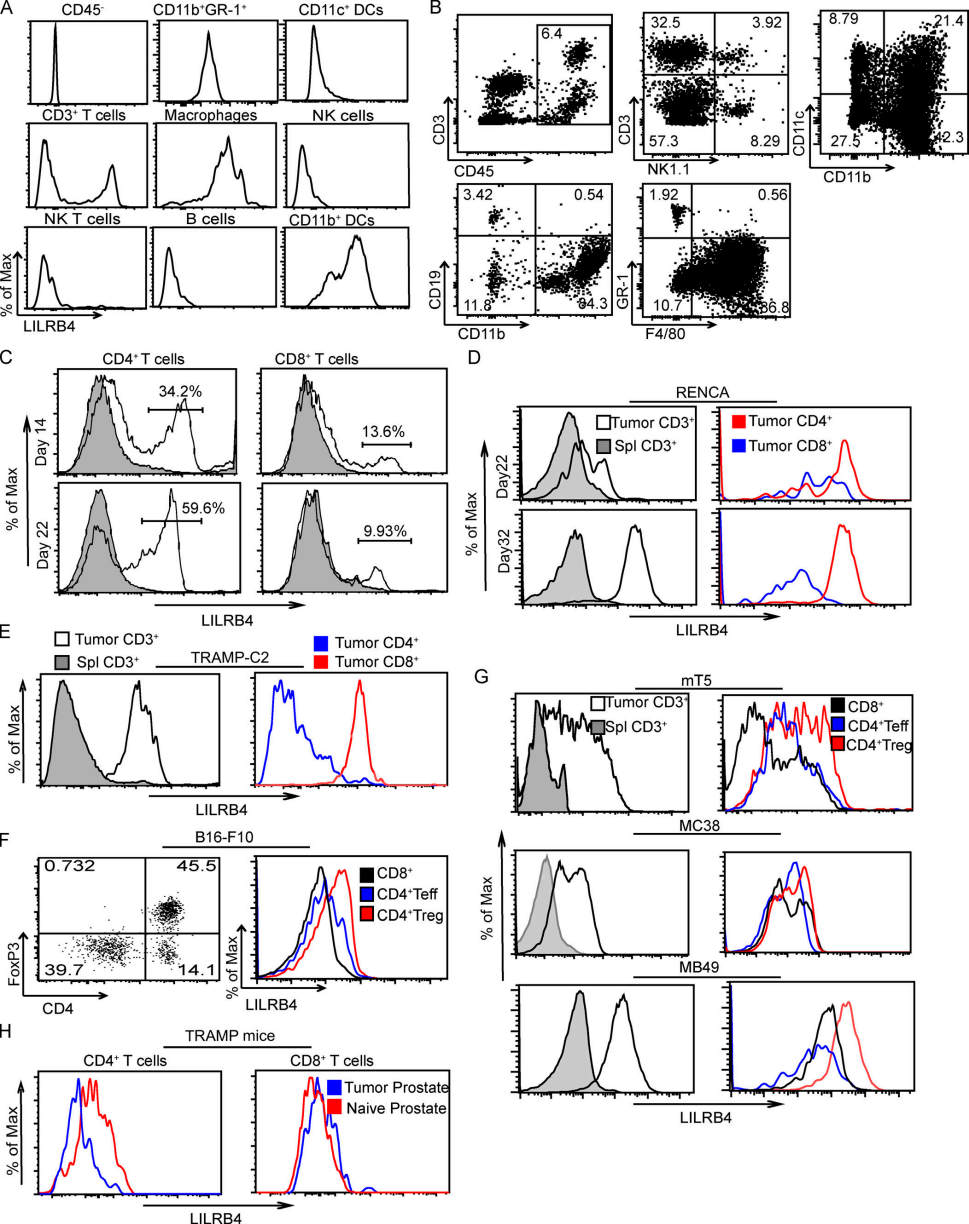
**Among CD45^+^ cells, LILRB4 is expressed largely on macrophages and Treg cells.** Mice were challenged with 3 × 10^5^ B16/F10 cells, 2 × 10^5^ RENCA cells, 1 × 10^6^ TRAMP-C2 cells, 1 × 10^5^ mT5 cells, 3 × 10^5^ MC38 cells, and 2 × 10^5^ MB49 cells on their right flanks subcutaneously, other than B16/F10, which was injected intradermally. Tumors and spleens from tumor-challenged mice were dissected on two different time points for B16/F10 (days 14 and 22) and RENCA (days 22 and 32) after tumor challenge. For the remainder, tumors were dissected when the tumor grew to 1,000 mm^3^. Tumor-infiltrating cells and splenic cells were isolated and stained with indicated antibodies as described in Materials and methods and run on flow cytometer. **(A)** LILRB4 expression on various cell types on tumor-infiltrating cells on day 22 after B16/F10 tumor challenge. **(B)** Dot plots showing gating strategy for individual cell types in B16/F10 tumor at day 22 after tumor challenge. **(C)** Histogram plots showing expression of LILRB4 on CD8^+^ and CD4^+^ T cells on days 14 and 22 after B16/F10 tumor challenge. **(D)** Histogram plots showing LILRB4 expression on splenic CD3^+^ T cells and tumor-infiltrating CD3^+^, CD4^+^, and CD8^+^T cells on days 22 and 32 after RENCA tumor challenge. **(E)** Histogram plots showing LILRB4 expression on splenic CD3^+^ T cells and tumor-infiltrating CD3^+^, CD4^+^, and CD8^+^T cells after TRAMP-C2 tumor challenge. **(F)** Dot plot showing gating scheme as cells are gated on CD3^+^ T cells (left) and histogram plot showing LILRB4 expression on tumor-infiltrating CD4^+^ Teff cells, CD4^+^ Treg cells, and CD8^+^T cells. **(G)** Histogram plots showing LILRB4 expression on splenic CD3^+^ T cells and tumor-infiltrating CD3^+^, CD4^+^ Teff, CD8^+^ T, and Treg cells after tumor challenge as indicated. **(H)** Histogram plots showing LILRB4 expression on prostate-infiltrated CD4^+^ and CD8^+^ T cells of TRAMP^+^ and littermate WT control mice. Data are representative of two or three independent experiments with five to seven mice in each experiment.

We challenged mice with B16/F10 cells and analyzed resulting tumors by flow cytometry with a pan-immune cell panel. LILRB4 was expressed on most tumor-infiltrating immune cell types, but the highest expression was on CD3^+^ T cell and CD11b^+^ cells. Within CD11b^+^ cells, LILRB4 was expressed on both F4/80^+^ macrophages and CD11b^+^ GR-1^+^ neutrophils. The expression of LILRB4 on CD11c^+^ DCs, NK cells, and NKT cells was not very high; tumor-infiltrating B cells did not express LILRB4 (Fig. S1, A and B). Within CD3^+^ T cells, the expression of LILRB4 was higher on CD4^+^ T cells than CD8^+^ T cells (Fig. S1 C). The expression of LILRB4 increased on CD4^+^ T cells with tumor progression (day 22 versus day 14 tumors) after B16/F10 challenge (Fig. S1 C). Similarly, we found the expression of LILRB4 was higher on CD4^+^ T cells than on CD8^+^ T cells within tumor-infiltrating CD3^+^ T cells in the mouse renal carcinoma model RENCA. There was also an increase in LILRB4 expression on CD3^+^ T cells with tumor progression (day 22 vs. day 32 tumor) in the RENCA tumor model (Fig. S1 D). We found a similar pattern of expression of LILRB4 in the TRAMP-C2 prostate cancer model, where the expression of LILRB4 was higher on CD4^+^ T cells than on CD8^+^ T cells (Fig. S1 E). To further analyze the expression pattern of LILRB4 within tumor-infiltrating T cell subsets, we analyzed LILRB4 expression in various murine tumor models, including B16/F10, pancreatic tumor model mT5, colon carcinoma model MC38, and bladder cancer model MB49. We also observed LILRB4 expression exclusively on tumor-infiltrating immune cells and not on splenic immune cells. Among T cells, LILRB4 was expressed at higher levels on Treg cells than on CD4^+^ effector T cells (Teff cells) or CD8^+^ T cells (Fig. S1, F and G). Because these nonspontaneous tumor models do not recapitulate the full process of oncogenesis, we decided to analyze LILRB4 expression in TRAMP mice, a spontaneous model of prostate cancer, where we compared the expression of LILRB4 on naive prostate to the TRAMP mice prostate. We observed elevated expression of LILRB4 on CD4^+^ T cells from TRAMP mice prostate compared with littermate WT mice prostate, but the expression on CD8^+^ T cells was the same (Fig. S1 H). These data suggest that LILRB4 is a potential target for tumor immunotherapy, as it is expressed on tumor-infiltrating immune cells of most tumor types and, most importantly, its expression is restricted to the TME.

### LILRB4 expression analysis of tumor-infiltrating T cells using cytometry by time of flight (CyTOF)

**Figure 2.**
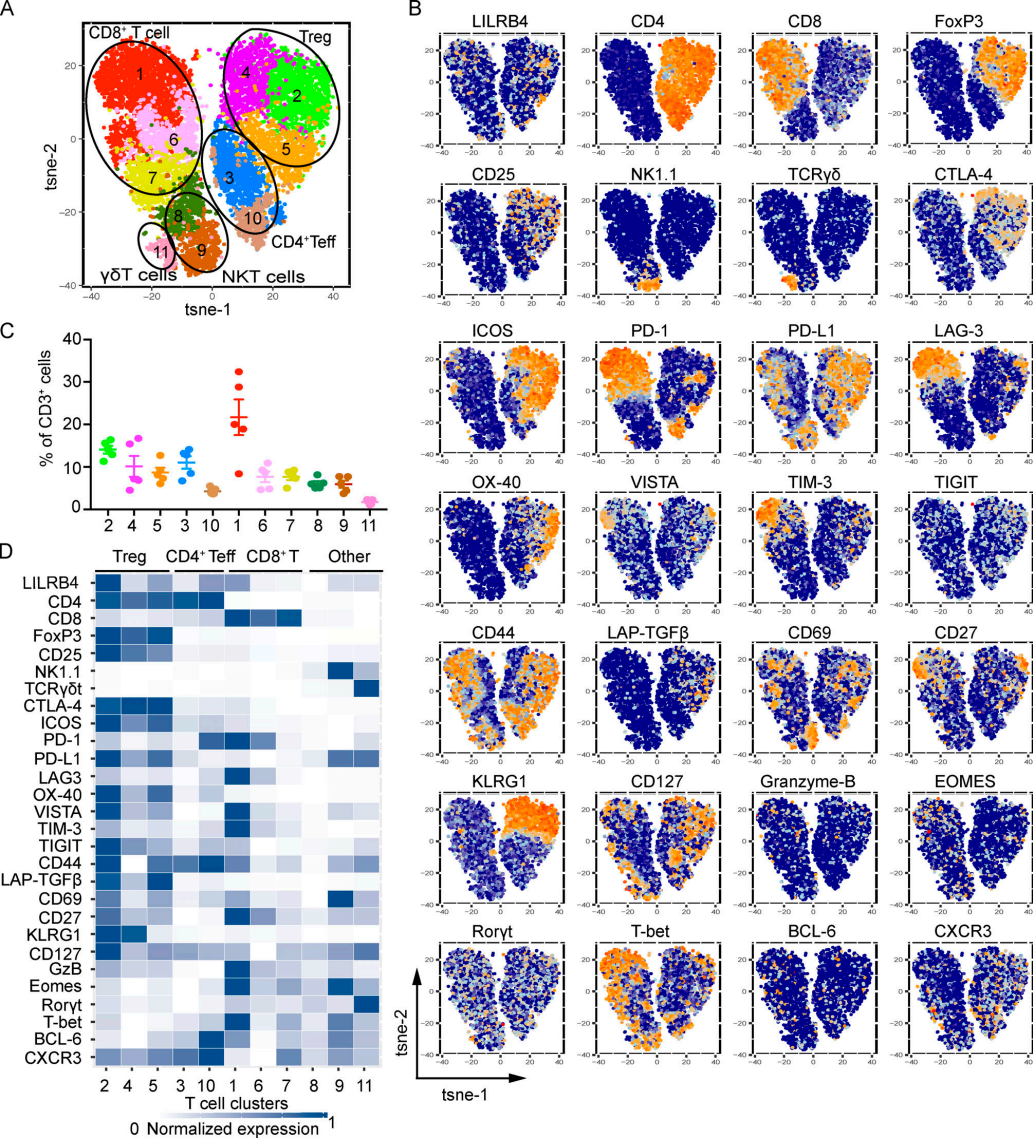
**T cell–expressed LILRB4 is largely on Treg cells and exhausted CD8^+^ T cells.** Mice were challenged with MC38 tumors, and when tumors grew to 1,000 mm^3^, mice were sacrificed and tumors were isolated. Tumor-infiltrating cells were isolated and stained with indicated CyTOF antibodies as described in Materials and methods and run on Helios. **(A)** t-SNE plot of MC38-infiltrating T cells overlaid with color-coded clusters. **(B)** t-SNE plot of infiltrating T cells overlaid with the expression of selected markers. **(C)** Frequency of clusters displayed on a per-mouse basis. Cluster numbers are indicated on the x-axis. **(D)** Heatmap displaying normalized marker expression of each cluster. Data are representative of three experiments with four to six mice in each experiment.

**Figure S2.**
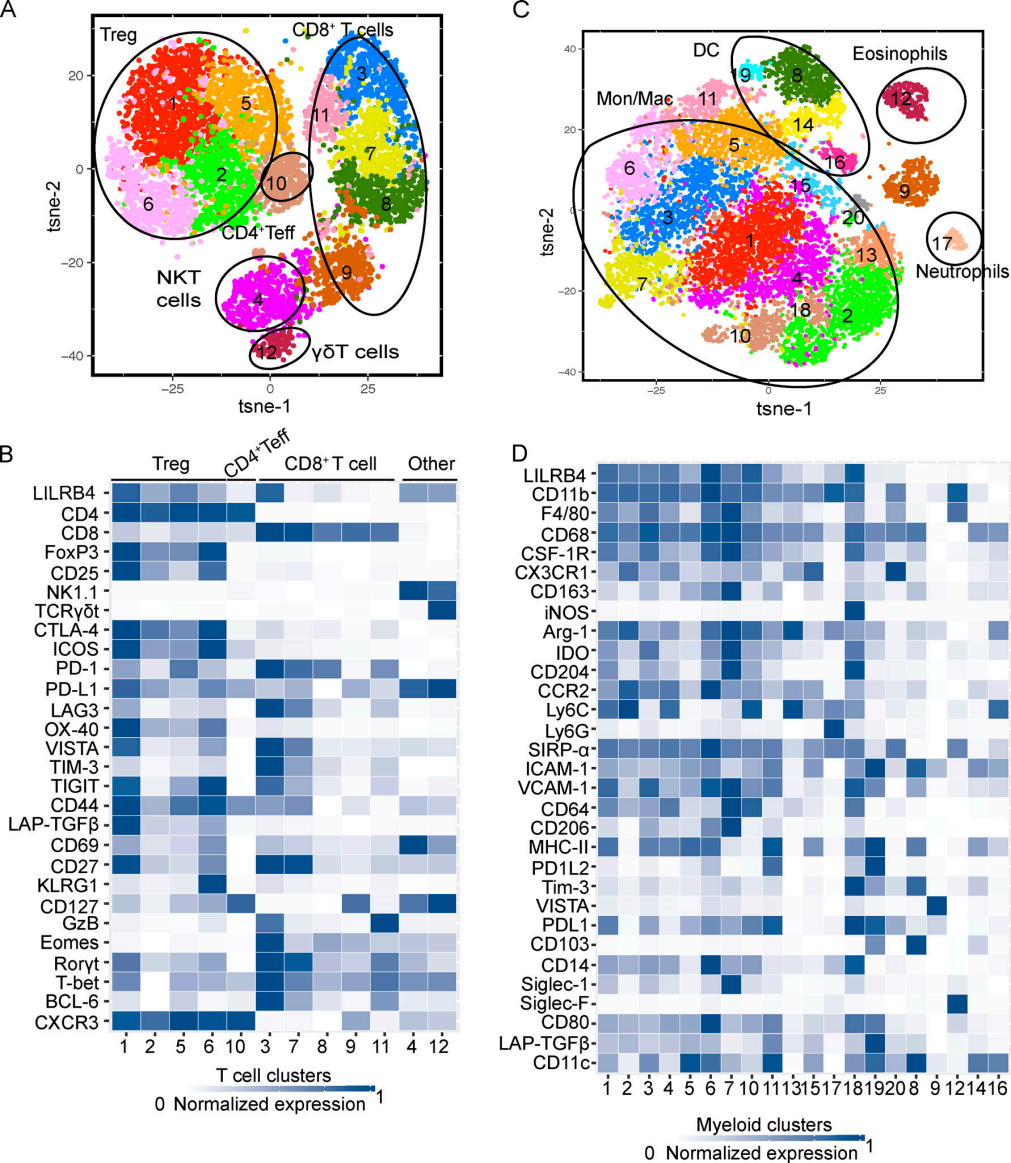
**LILRB4 is expressed largely on Treg cells, exhausted CD8^+^ T cells, and CD11b^+^ TAMs in the B16/F10 tumor model.** Mice were challenged with 3 × 10^5^ B16/F10 tumor cells, and when tumors grew to 1,000 mm^3^, mice were sacrificed and tumors were isolated. Tumor-infiltrating cells were isolated and stained with indicated CyTOF antibodies as described in Materials and methods and run on Helios. **(A)** t-SNE plot of MC38 infiltrating T cells overlaid with color-coded clusters. **(B)** Heatmap displaying normalized marker expression of each T cell cluster. **(C)** t-SNE plot of MC38 infiltrating CD45^+^CD3^−^ cells overlaid with color-coded clusters. **(D)** Heatmap displaying normalized marker expression of each cluster. Data are representative of three experiments with four to six mice in each experiment.

To comprehensively profile the intratumoral T cell populations expressing LILRB4, we used mass cytometry and a well-validated, data-driven unsupervised clustering approach on cells from MC38 and B16/F10 tumors. We designed a staining panel with T cell subset marker antibodies (CD4, CD8, and FoxP3), NK1.1, TCR-γδ, functional molecules such as granzyme B, transcription factors such as Eomes, and various inhibitory receptors. We generated a high-resolution map of phenotypically defined tumor-infiltrating T cell populations using unsupervised clustering. 11 clusters were identified in MC38 tumor model with relative frequency greater than 0.5%, which included three Treg cell clusters, two CD4^+^ Teff clusters, three CD8^+^ T cell clusters, and three other clusters (two NKT cells and one γδ T cell clusters; Fig. 2, A–D). Among CD3^+^ T cells, LILRB4 expression was highest on Treg cells, which were divided into three clusters, KLRG1^high^ ICOS^high^ TGFβ^high^, KLRG1^high^ ICOS^+^ TGFβ^low^, and KLRG1^low^ ICOS^high^ TGFβ^high^. The highest expression of LILRB4 was on the KLRG1^high^ ICOS^high^ TGFβ^high^ Treg cell cluster, and the lowest expression was on the KLRG1^low^ ICOS^high^ TGFβ^high^ cluster. There were two clusters of CD4^+^ Teff cells, PD-1^high^ LAG3^high^ and PD-1^−^ LAG3^−^; the expression of LILRB4 was higher on PD-1^high^ LAG3^high^ CD4^+^ Teff cells. Among three distinct CD8^+^ T cell clusters, LILRB4 was expressed only on the PD-1^high^ LAG3^high^ Tim-3^high^ cluster (Fig. 2 D). These results further confirm the association of LILRB4 with inhibitory receptors such as PD-1 and LAG-3. CD4^+^ T cells and CD8^+^ T cells expressing these inhibitory receptors are exhausted T cells, as chronic stimulation up-regulates these inhibitory receptors (Crespo et al., 2013; Schietinger and Greenberg, 2014). We also profiled B16/F10 tumors using the same panel and observed 12 cell clusters, which included four Treg cell clusters, one CD4^+^ Teff cell cluster, five CD8^+^ T cell clusters, and two other clusters (one NKT cells and one γδ T cell cluster). Similar to MC38 tumors, we found LILRB4 expression largely on CD4^+^ Treg cells; the expression of LILRB4 on CD4^+^ Teff cells was very low. Among CD8^+^ T cell clusters, the highest expression of LILRB4 was on PD-1^high^ LAG3^high^ Tim3^high^ CD8^+^ T cells similar to what we observed in MC38 tumors (Fig. S2, A and B). These results confirm that among T cell subsets, LILRB4 is expressed mostly on Treg cells, and the expression on CD4^+^ and CD8^+^ T cells is associated with expression of other inhibitory receptors.

### LILRB4 expression analysis of tumor-infiltrating myeloid cells by CyTOF

**Figure 3.**
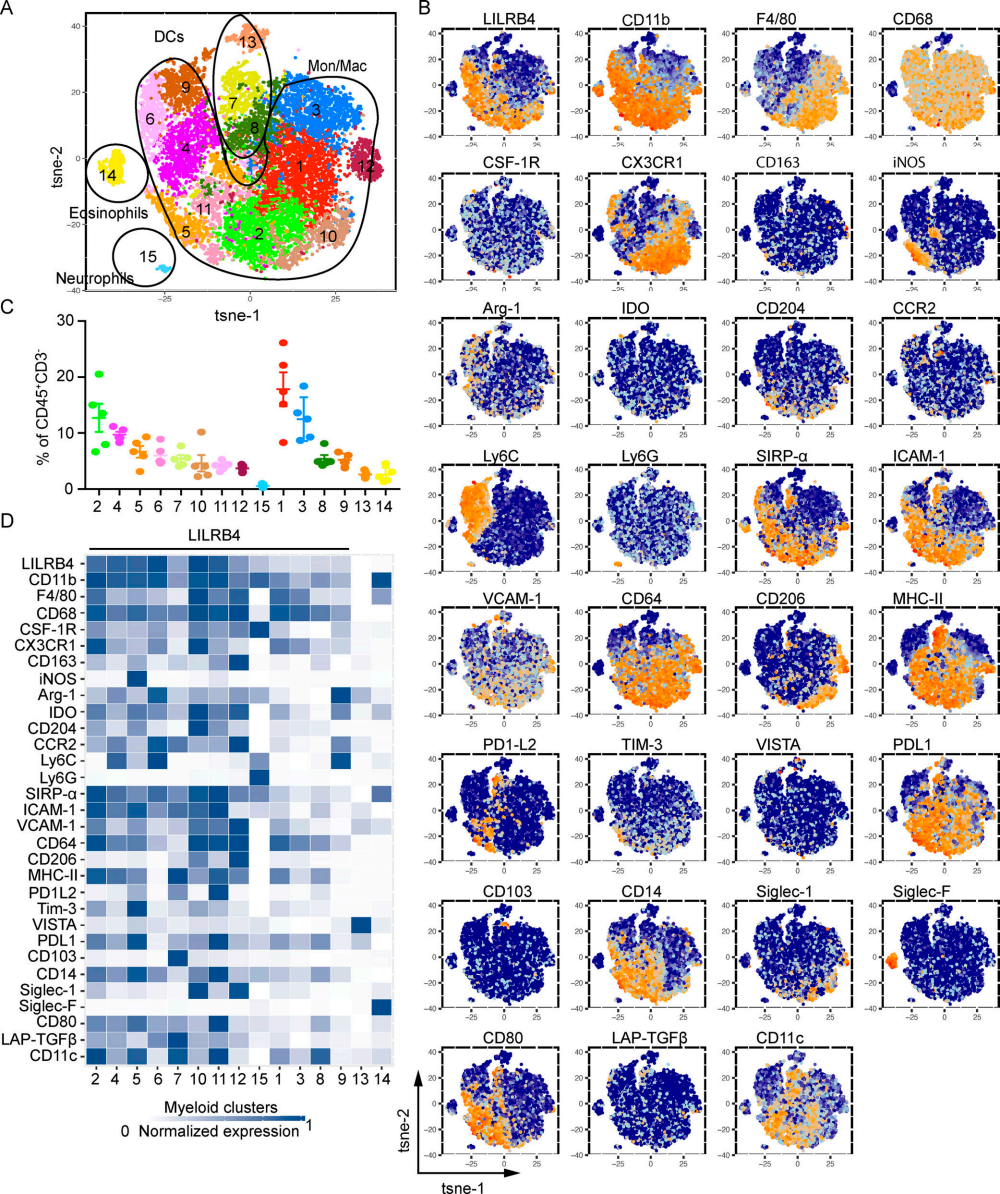
**Myeloid-expressed LILRB4 is largely on CD11b^+^ TAMs and associated with markers of suppressive TAMs.** Mice were challenged with MC38 tumors, and when tumors grew to 1,000 mm^3^, mice were sacrificed and tumors were isolated. Tumor-infiltrating cells were isolated and stained with indicated CyTOF antibodies as described in Materials and methods and run on Helios. **(A)** t-SNE plot of MC38-infiltrating CD45^+^CD3^−^ cells overlaid with color-coded clusters. **(B)** t-SNE plot of infiltrating CD45^+^CD3^−^ cells overlaid with the expression of selected markers. **(C)** Frequency of clusters displayed on a per-mouse basis. Cluster numbers are indicated on the x-axis. **(D)** Heatmap displaying normalized marker expression of each cluster. Data are representative of three experiments with four to six mice in each experiment.

To comprehensively profile the LILRB4 expression on intratumoral myeloid cells, we used a myeloid-focused CyTOF panel and MC38 tumor model. Analysis of this panel revealed 15 myeloid cell clusters with frequency greater than 0.5%, including multiple populations of monocytes/macrophages (clusters 1–6 and 9–12), DCs clusters (clusters 7, 8, and 13), an eosinophil cluster (cluster 14), and a neutrophil cluster (cluster 15; Fig. 3, A–D). LILRB4 was expressed on most clusters, other than clusters 13 and 14. LILRB4 was expressed on all monocyte/macrophage clusters, but the expression levels were variable among clusters. We found three clusters that had the highest expression of LILRB4: cluster 6 (CD11b^+^ F4/80^low^ Arg-1^high^ CCR2^high^ CX_3_CR1^+^ Ly6C^high^ cluster), cluster 10 (CD11b^+^ F4/80^high^ Arg1^+^ IDO^high^ CD204^high^ CD64^+^ CX_3_CR1^high^ CD206^+^ CCR2^+^ Ly6C^+^ cluster), and cluster 11 (CD11b^+^ F4/80^low^ CD68^high^ CX_3_CR1^low^ CD163^+^ Arg1^+^ IDO^+^ CD204^+^ CD64^+^ PD-L1^high^ PD1L2^high^; Fig. 3 D). Arg-1, CX_3_CR1, CD206, CD163, and IDO are the markers that have been shown to be associated with TAMs with an immunosuppressive phenotype, suggesting LILRB4 expression on suppressive TAMs (Biswas and Mantovani, 2010; Gubin et al., 2018; Murray et al., 2014; Qian and Pollard, 2010). We used the same panel to analyze myeloid clusters in B16/F10 tumor. There were 20 clusters with multiple monocyte/macrophage clusters (clusters 1–7, 9–11, 13, 15, and 18), multiple DCs clusters (clusters 8, 14, 16, and 19), an eosinophil cluster (cluster 12), and a neutrophil cluster (cluster 17; Fig. 2 C). Similar to MC38, we found that LILRB4 was highly expressed on monocyte/macrophage clusters, with variable expression on different subsets. There were a few clusters that did not express LILRB4 or expressed it at very low levels, such as clusters 8, 9, 12, 14, 16, 19, and 20. The highest expression of LILRB4 was on cluster 6 (CD11b^+^ F4/80^low^ Arg-1^+^ IDO^+^ CCR2^high^ CX3CR1^+^ cluster), cluster 10 (CD11b^+^ F4/80^+^ CD68^high^ Arg1^+^ Ly6C^+^ cluster), and cluster 18 (CD11b^+^ F4/80^+^ CD68^+^ iNOS2^high^ CD204^high^ Arg1^+^ Tim3^high^ PD-L1^high^ Ly6C^+^ cluster; Fig. S2 D). Similar to the MC38 tumor, LILRB4 is expressed on most subsets of macrophages, with higher expression on TAMs with suppressive phenotype. This was further confirmed by expression analysis of LILRB4 on in vitro skewed M1 and M2 bone marrow–derived macrophages (BMDMs) by flow cytometry. We found that LILRB4 expression was higher on M2-skewed BMDMs compared with M1-skewed BMDMs, although M1-skewed BMDMs also expressed LILRB4 on the surface (data not shown).

**Figure S3.**
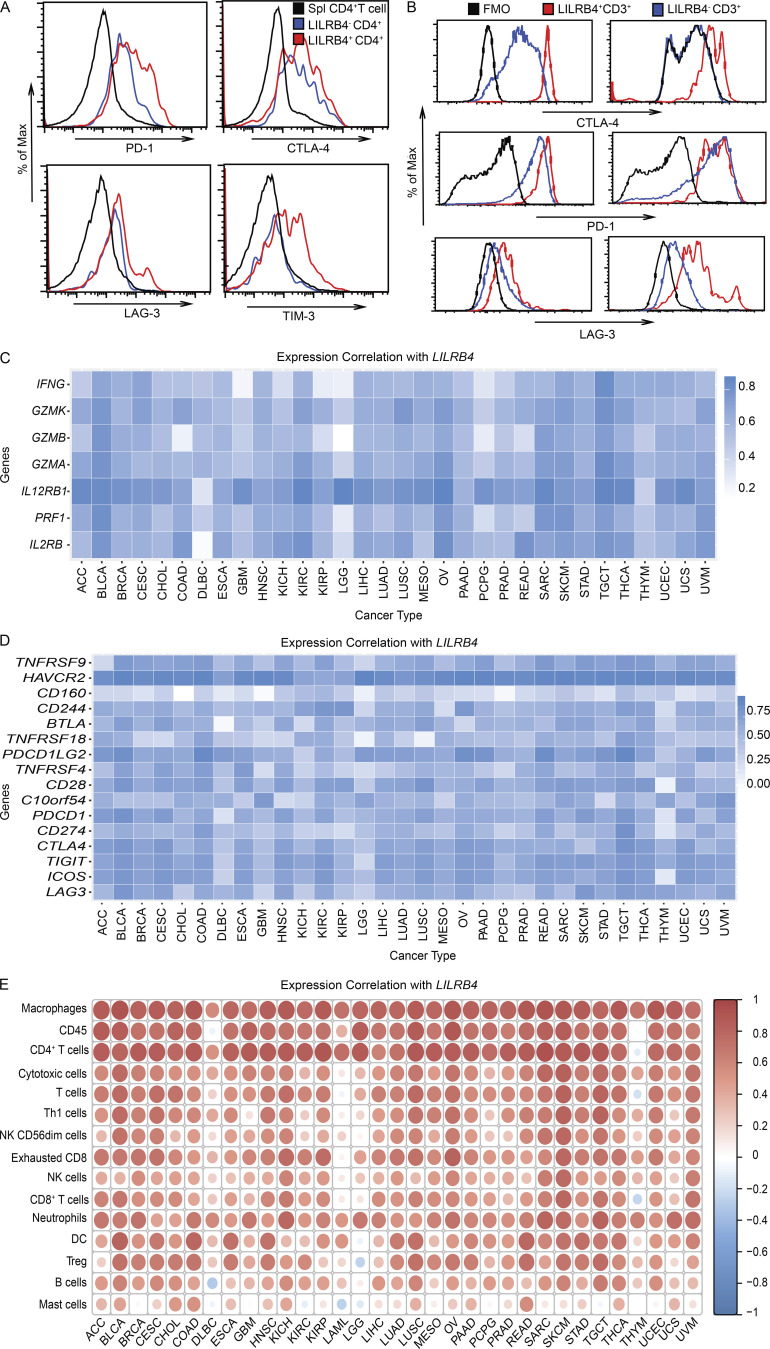
**LILRB4 expression is correlated with other inhibitory receptor and immune cell infiltrates. (A)** Mice were challenged with 3 × 10^5^ B16/F10 cells on their right flanks intradermally. Tumors and spleens were dissected when tumors grew to 1,000 mm^3^, digested, and then tumor-infiltrating cells and splenic cells were isolated and stained with indicated antibodies as described in Materials and methods and run on flow cytometer. Histogram plots showing expression of various inhibitory receptors on tumor-infiltrated LILRB4^+^ and LILRB4^−^ CD4^+^ T cells whereas splenic naive CD4^+^ T cells act as negative control. **(B)** Tumor from melanoma patient was digested and stained with indicated antibodies as described in Materials and methods. Histogram plots showing expression of various inhibitory receptors on LILRB4^+^ and LILRB4^−^ CD3^+^ T cells. Black lines are FMO (fluorescence minus one). **(C–E) **Bioinformatics analysis of correlation of *LILRB4* expression in data from TCGA database with functional molecules (C), various inhibitory/immune receptors (D), and immune cells (E). ACC, adrenocortical carcinoma; BLCA, bladder urothelial carcinoma; BRCA, breast invasive carcinoma; CESC, cervical squamous cell carcinoma; CHOL, cholangiocarcinoma; COAD, colon adenocarcinoma; DLBC, lymphoid neoplasm diffuse large B cell lymphoma; ESCA, esophageal carcinoma; GBM, glioblastoma multiforme; HNSC, head and neck squamous cell carcinoma; KICH, kidney chromophobe; KIRC, kidney renal clear cell carcinoma; KIRP, kidney papillary cell carcinoma; LAML, acute myeloid leukemia; LGG, low grade glioma; LIHC, liver hepatocellular carcinoma; LUAD, lung adenocarcinoma; LUSC, lung squamous cell carcinoma; MESO, mesothelioma; OV, ovarian cancer; PAAD, pancreatic adenocarcinoma; PCPG, pheochromocytoma and paraganglioma; PRAD, prostate adenocarcinoma; READ, rectum adenocarcinoma; SARC, sarcoma; SKCM, skin cutaneous melanoma; STAD, stomach adenocarcinoma; TGCT, testicular germ cell tumors; THCA, thyroid carcinoma; THYM, thymoma; UCEC, uterine corpus endometrial carcinoma; UCS, uterine carcinosarcoma; UVM, uveal melanoma.

Similar to our CyTOF results, LILRB4 expression on T cells was highly correlated with other inhibitory receptors in both murine tumors and human cancer patient samples analyzed by flow cytometry (Fig. S3, A and B). We analyzed correlation of *LILRB4* gene expression with various functional molecules, including serine proteases such as granzymes (*GZMK*, *GZMB*, and *GZMA*), perforin (*PRF1*), cytokine like *IFNG*, and cytokine receptors *IL12RB1* and *IL2RB* in data from TCGA database. *LILRB4* was highly correlated with *IL12RB1* and *IL2RB* across the various cancer types. It also had high correlation with granzymes and perforin, especially in skin cutaneous melanoma and bladder cancer (Fig. S3 C). We also found *LILRB4* expression to be highly correlated with other inhibitory molecules, specifically *PDCD1* (*PD1*) and *HAVCR2* (*TIM3*), in different tumor types (Fig. S3 D). Correlation analysis of *LILRB4* with various cell subsets in different cancer patients suggests that *LILRB4* is expressed in most tumor-infiltrating immune cell types. Among the different cell types, *LILB4* expression is most correlated with tumor-infiltrating macrophages and CD4^+^ T cells (Fig. S3 E). This agrees with our analysis of LILRB4 expression in murine models and tumor patients.

### *Lilrb4* expression analysis by scRNA-seq

**Figure 4.**
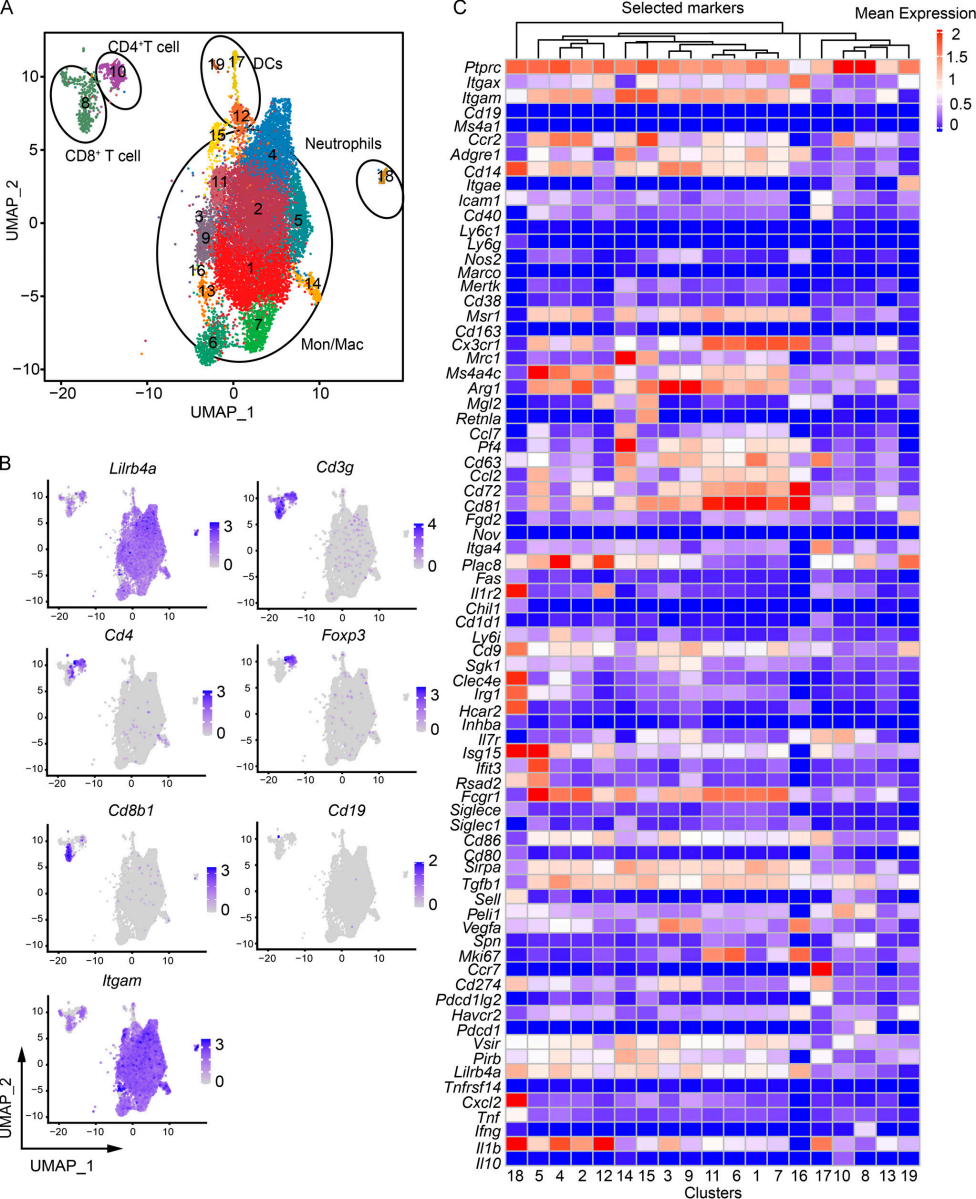
**scRNA-seq analysis of *Lilrb4* expression in tumor-infiltrated cells. **Mice were challenged with MC38 tumors, and when tumors grew to 1,000 mm^3^, mice were sacrificed and tumors were isolated. Tumor-infiltrating cells were isolated and stained with CD45 antibody for sorting by FACS, and a 10X library was prepared and analyzed as described in Materials and methods. **(A)** UMAP graph showing the clusters and annotation. **(B)** UMAP plots showing the expression of selected markers. **(C) **Heatmap displaying selected marker expression for each cluster. Data shows computationally combined two independent experiments with four to six mice in each experiment.

We used scRNA-seq to analyze the *Lilrb4* expression in tumor-infiltrating CD45^+^ immune cells. To that end, tumors were isolated from MC38 tumor-challenged mice, digested, and live CD45^+^ cells were FACS sorted. 8,000–10,000 cells were targeted with a coverage of ∼30,000–50,000 mean reads per cell (Table S1). Data from two experiments were computationally pooled for analysis, and 20 clusters that were *ptprc-*positive (CD45^+^) were used for further analysis (Fig. 4 A). These clusters were defined manually on the basis of known cell markers and by using SingleR pipeline with ImmGen database as a reference dataset (Aran et al., 2019). Out of these clusters, there were 13 monocytes/macrophages clusters (clusters 1–7, 9, 11, and 13–16), three DCs clusters (clusters 12, 17, and 19), two T cell clusters (clusters 8 and 10), and one neutrophil cluster (cluster 18; Fig. 4, A and B). The *Lilrb4* was promiscuously expressed in all cell types and associated with *Cd3g* (CD3^+^ T cells), *Itgam *(CD11b^+^ cells), and *Itgax *(CD11c^+^ cells). There was no *Cd19*-expressing cluster out of these clusters which suggests no tumor-infiltrating B cell population. Among *Cd3g-*positive T cell clusters, *Lilrb4* was expressed in both *Cd4*-negative (cluster 10) and *Cd8b1*-positive cluster (cluster 8). However, *Lilrb4* expression was relatively higher in monocytes/macrophages and neutrophil clusters than other cell type clusters. Among the macrophage clusters, *Lilrb4* was expressed in all macrophage clusters with relatively higher expressions in clusters 4, 9, 14, 15, and 16. Cluster 4 was positive for *Ccr2*, *Msr1*, *Ms4a4c*, *Arg1*, and *Fcgr1*. Cluster 9 had high expression of *Arg1*,**along with expression of *Msr1*(Cd204) and *Ms4a4c*. Cluster 15 was positive for *Ccr2*, *Mrc1*(Cd206), *Arg1* *Mgl2*, and *Retnla*, whereas cluster 16 displayed expression of *Msr1*, *Cx3cr1*, *Mgl2*, and *Cd72*. Expression of these markers is associated with M2-type macrophages, which further confirms our CyTOF data that *Lilrb4* is highly expressed on M2-type macrophages.

### Anti-LILRB4 antibody treatment prolongs survival in tumor-bearing mice

**Figure 5.**
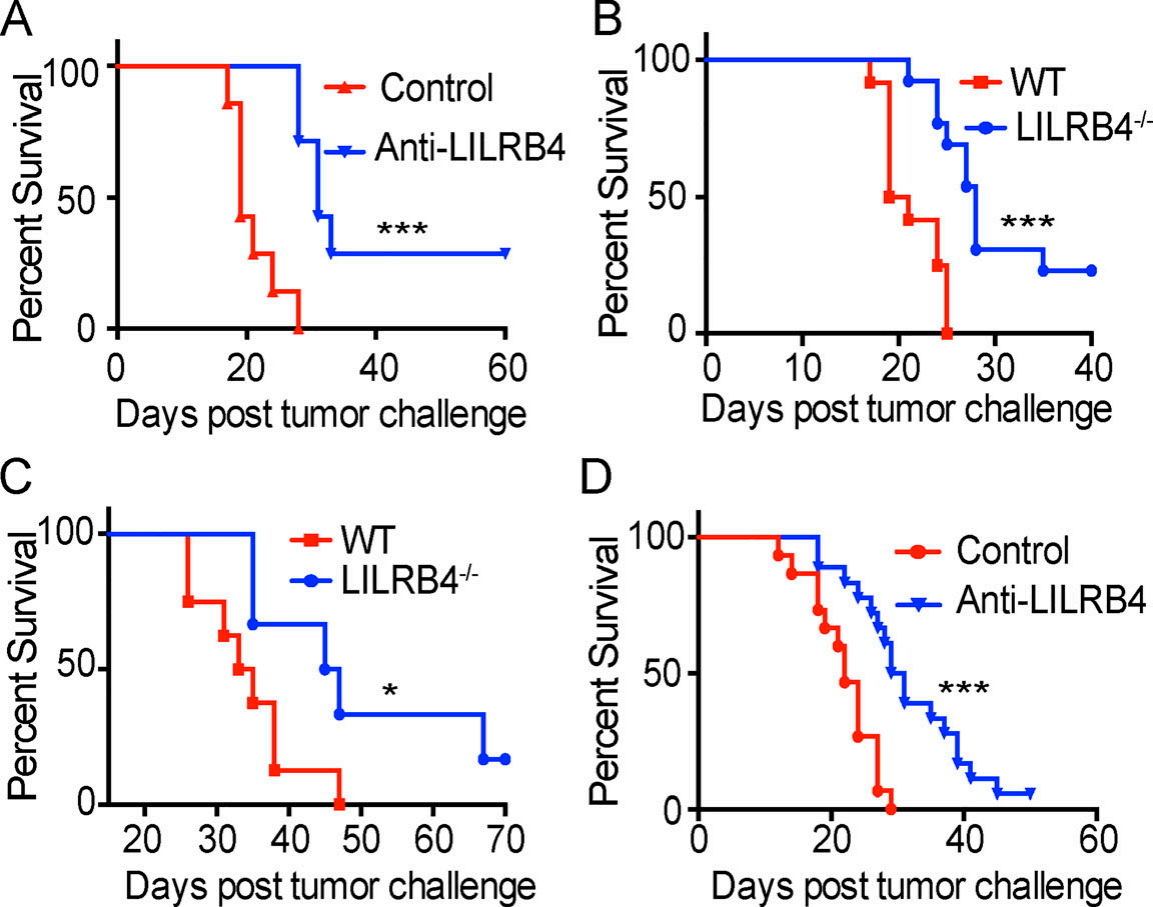
**Tumor-challenged LILRB4^−/−^ mice or WT mice given anti-LILRB4 treatment survive longer than controls.** **(A)** Mice were challenged with 3 × 10^5^ B16/F10 cells on right flanks and were given intratumoral injection of polyclonal anti-LILRB4 antibody or isotype control. Survival of mice in each treatment group is shown. **(B)** WT and LILRB4^−/−^ mice were challenged with 3 × 10^5^ B16/F10 cells. Survival of mice in each group is shown. **(C)** WT and LILRB4^−/−^ mice were challenged with 10^5^ mT5 cells subcutaneously. Survival of mice in each group is shown. **(D)** Mice were challenged with the MC38 tumor model subcutaneously on the right flank and were given an intraperitoneal injection of anti-LILRB4 monoclonal antibody on days 3, 6, 9, and 12. Survival of mice in each group is shown. Data are representative of two or three independent experiments with 5–10 mice per group. *, P < 0.05; ***, P < 0.001 (Mantel–Cox test).

**Figure S4.**
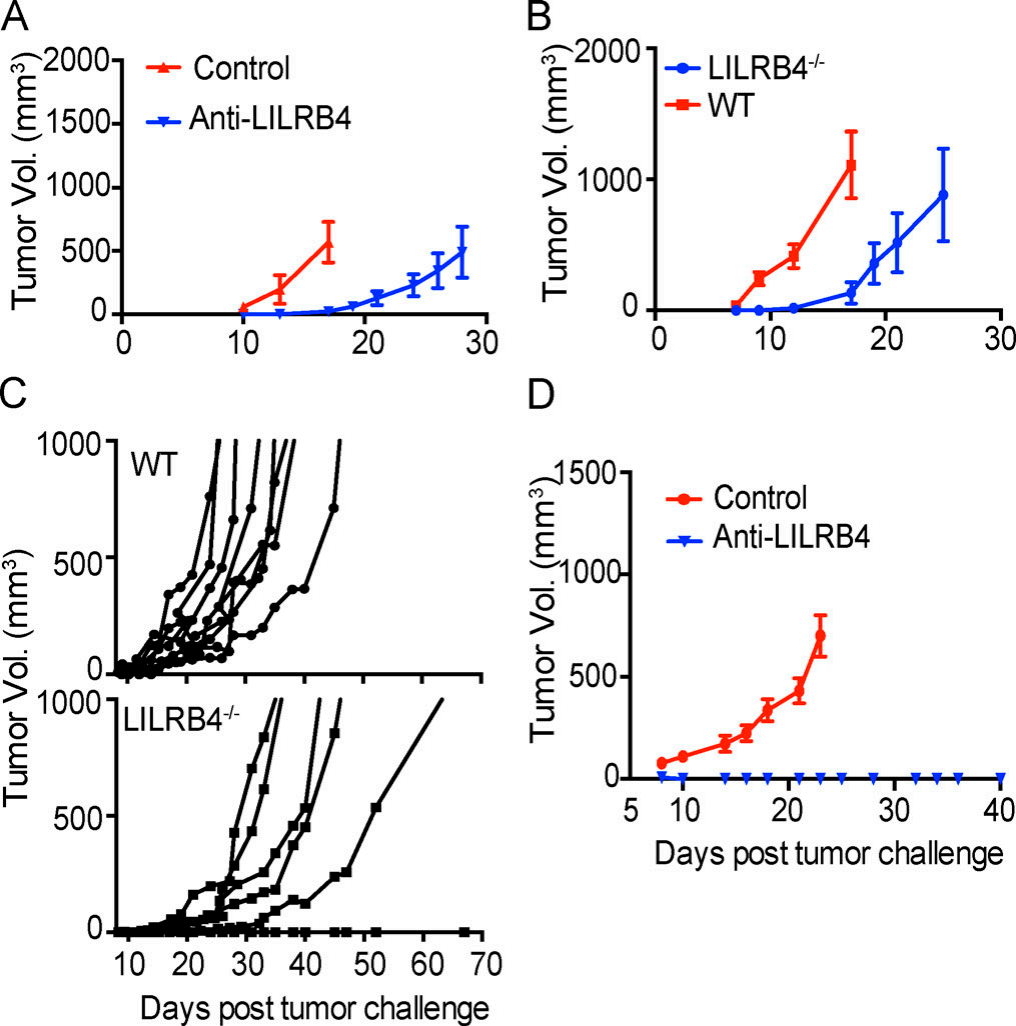
**Tumor-challenged LILRB4^−/−^ mice or WT mice given anti-LILRB4 treatment have reduced tumor burden compared with controls during primary challenge and rechallenge.** **(A)** Average tumor volume in mice that were challenged with 3 × 10^5^ B16/F10 cells on right flanks and were given intratumoral treatment of polyclonal anti-LILRB4 antibody or isotype control as shown on days 3, 6, 9, and 12. **(B)** Average tumor burden in LILRB4^−/−^ and WT mice that were challenged with 3 × 10^5^ B16/F10 cells. **(C)** Individual tumor burden in WT and LILRB4^−/−^ mice that were challenged with 10^5^ mT5 cells subcutaneously. **(D)** Anti-LILRB4 antibody induces immunological memory. Mice that survived primary MC38 challenge and had been earlier treated with anti-LILRB4 antibody were rechallenged with 1.5 × 10^6^ MC38 cells and left untreated. The blue line represents the average tumor burden of memory mice whereas red line represents naive mice with no earlier tumor challenge or treatment, which served as a control. Data are representative of two or three independent experiments with 5–10 mice per group.

To test the hypothesis that LILRB4 is a critical negative regulator of antitumor responses, we employed anti-LILRB4 polyclonal antibody in a tumor burden and survival experiment. This antibody was injected intratumorally on days 3, 6, 9, and 12 after mice were challenged with B16/F10 tumor. There was a decrease in tumor burden and an increase in survival in mice injected with this antibody compared with isotype antibody control (Fig. 5 A and Fig. S4 A). Almost 30% of mice that received anti-LILRB4 completely rejected tumors, and mice that did show tumor burden had delayed tumor growth. We next employed LILRB4^−/−^ mice, which were described earlier (Rojo et al., 2000), challenged them with B16/F10 tumor cells, and evaluated tumor burden and survival. There was a decrease in tumor burden and prolonged survival of tumor-challenged mice lacking LILRB4 (LILRB4^−/−^) compared with WT control (Fig. 5 B and Fig. S4 B). As we observed LILRB4 expression in various tumor models, we wanted to know if LILRB4 functions as a negative regulator of tumor immunity across multiple models. To this end, we challenged LILRB4^−/−^ and WT mice with mT5 tumor subcutaneously and measured tumor burden and survival. Similar to the B16F/10 tumor model, we observed a significant decrease in tumor burden and increase in survival in LILRB4^−/−^ mice compared with WT control (Fig. 5 C and Fig. S4 C).

Encouraged by our results with polyclonal antibody and LILRB4^−/−^ mice, we developed a monoclonal hamster antibody against LILRB4, and an antibody clone for the in vivo assay was selected as described in Materials and methods. C57BL/6J mice were challenged with MC38 tumor and then treated intraperitoneally with monoclonal anti-LILRB4 antibody on days 3, 6, 9, and 12. We found prolonged survival in mice injected with anti-LILRB4 antibody compared with isotype control antibody (Fig. 5 D). Immunological memory is an important aspect of immunotherapy that underlies durable antitumor responses. We sought to determine whether the anti-LILRB4 antibody produces immunological memory in treated mice. We pooled mice from different experiments that were given monoclonal anti-LILRB4 antibody in primary MC38 tumor challenge, and survived. These mice were rechallenged with a very high dose of the MC38 tumor without any further treatment. All previously antibody-treated mice completely cleared MC38 rechallenge and had 100% survival rate (Fig. S4 D). These data indicate that anti-LILRB4 antibody induces tumor antigen–specific immunity in treated mice.

### Loss of LILRB4 signaling increases expression of immune-related genes in TME

**Figure 6.**
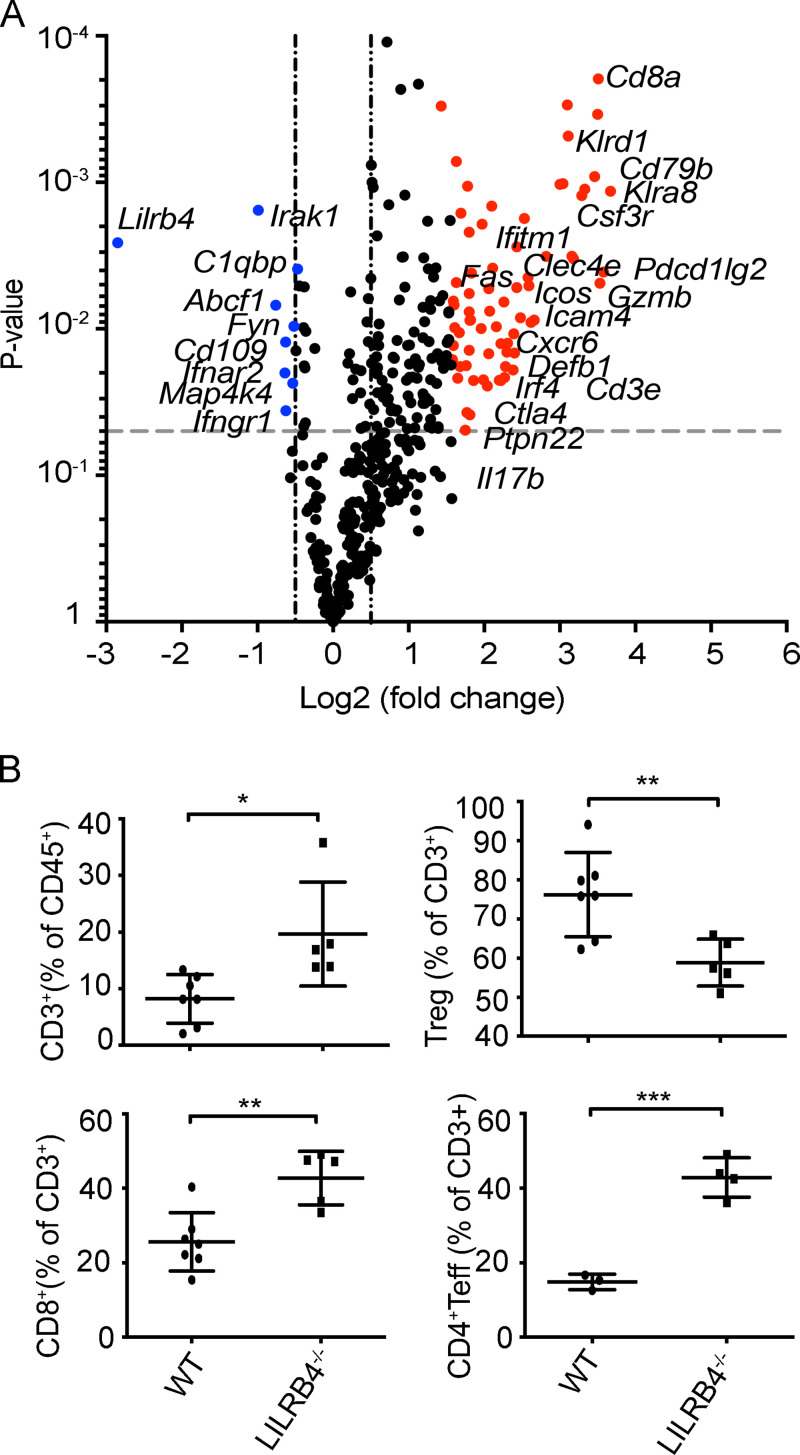
**Tumors in LILRB4^−/−^ mice have increased immune infiltrate.** **(A)** Volcano plot illustrates the log2 fold difference in gene expression (LILRB4^−/−^ versus WT) as determined by NanoString analysis in isolated tumor-infiltrating cells from mice challenged with B16/F10 cells. Top 25 genes up-regulated in LILRB4^−/−^ are colored red and down-regulated genes in blue. **(B)** Flow cytometry analysis of tumors isolated from LILRB4^−/−^ and WT mice challenged with 3 × 10^5^ MC38 tumor cells. Data are representative of two or three independent experiments with five to seven mice in each group. Error bars represent the mean ± SD. *, P < 0.05; **, P < 0.01; ***, P < 0.001 (Student’s *t* test).

We examined changes in gene expression in the TME caused by lack of LILRB4. Tumors were dissected from LILRB4^−/−^ and WT mice that were challenged with B16/F10 cells. mRNA was extracted from dissociated tumors; data were generated using NanoString technology and analyzed as described before (Sharma et al., 2019). A number of genes were up-regulated in tumors of LILRB4^−/−^ mice compared with WT controls (Fig. 6 A and Table S2). Table S2 shows the 25 genes with the most log2 fold change in the LILRB4^−/−^ group compared with the WT group. Many of these genes that were up-regulated in LILRB4^−/−^ tumors, such as *Cd3e*, *Cd8a*, and *Gzmb*, are associated with antitumor phenotypes. To determine whether these increased immune signatures in LILRB4^−/−^ mice observed by NanoString RNA analysis are present at the protein level, we analyzed tumor-infiltrating lymphocytes (TILs) from MC38 tumor–bearing mice by flow cytometry. We observed an increase in frequencies of CD3^+^ T cells, CD8^+^ T cells, and CD4^+^ Teff cells but a decrease in frequency of Treg cells in LILRB4^−/−^ mice (Fig. 6 B).

**Figure S5.**
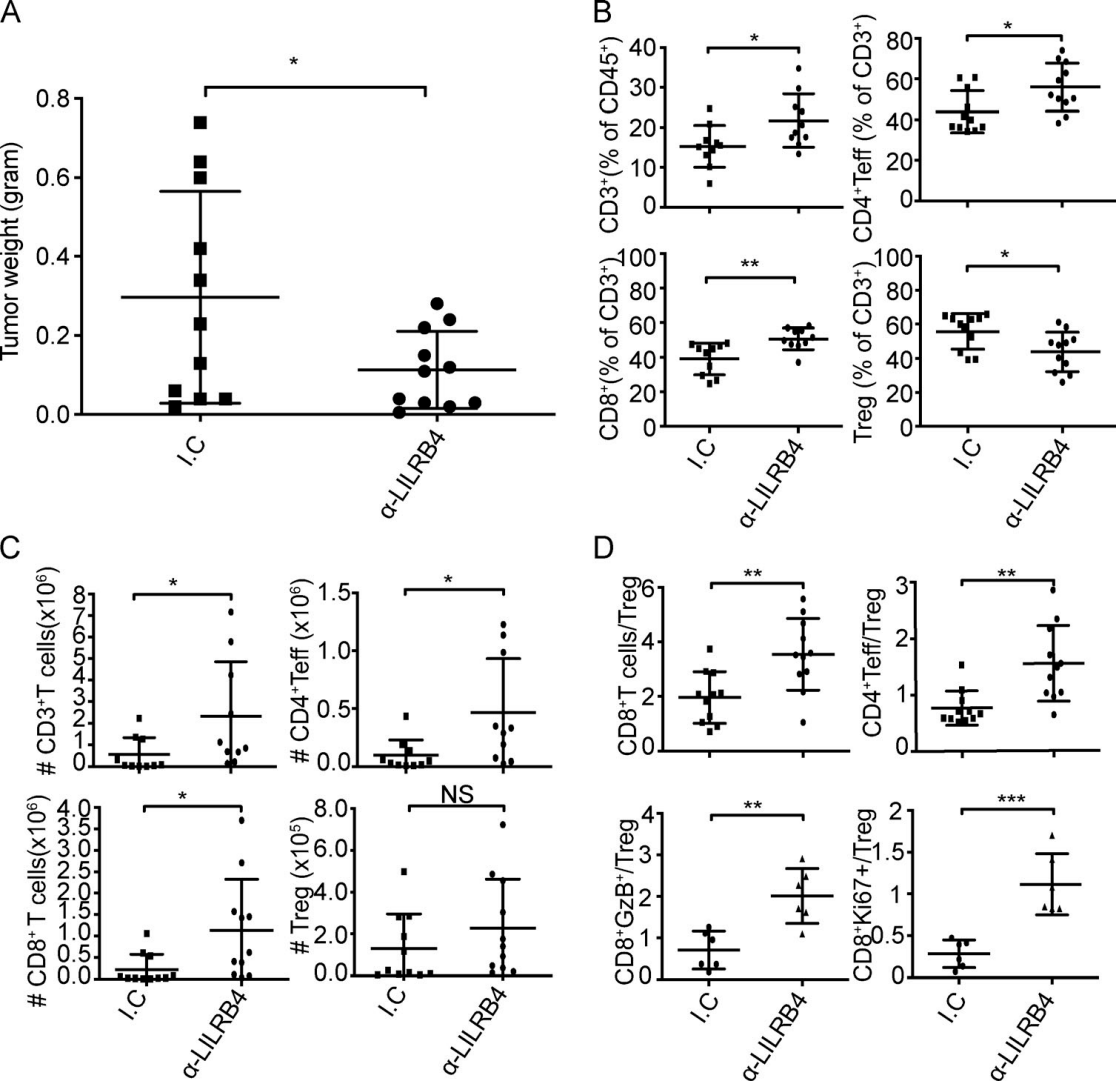
**Anti-LILRB4 treatment decreased tumor weight** **and increased T cell frequencies, and Teff/Treg cell ratios in the TME. **Mice were challenged with 6 × 10^5^ B16/F10 cells and given two doses of anti-LILRB4 on days 9 and 12 after tumor challenge. Tumors were dissected, and tumor weight was measured. Tumor cells were isolated and stained with indicated antibodies as described in Materials and methods. **(A and B)** Cumulative tumor weights of the indicated treatment groups (A); and cumulative frequencies of CD3^+^ T cells as percentages of CD45^+^ cells, CD8^+^ T cells as percentages of CD3^+^ T cells, CD4^+^ Teff cells, and Treg cells as percentages of CD4^+^ T cells (B). **(C)** These cell types’ respective densities as the cumulative absolute numbers of the cells per gram of tumor. **(D)** Cumulative CD8^+^ T cells/Treg cell, CD4^+^ Teff/Treg cell, CD8^+^GzB^+^/Treg cell, and CD8^+^Ki67^+^/Treg cell ratios in B16/F10 tumors on day 14 in each group. Data are cumulative of two or three independent experiments with five to seven mice in each group. Error bars represent the mean ± SD. *, P < 0.05; **, P < 0.01; ***, P < 0.001 (Student’s *t* test). I.C: Isotype control.

We assessed the functional effect of anti-LILRB4 antibody treatment on tumor-infiltrating T cells. Anti-LILRB4 antibody significantly decreased tumor weight compared with isotype control antibody (Fig. S5 A). Subsequently, cells were isolated from tumors, stained with antibodies, and run on a flow cytometer. We analyzed the percentages and population numbers of each subset of T cells within tumors. There was a significant increase in percentages and population of CD3^+^ T cell population within tumors in the anti-LILRB4–treated animal group compared with isotype control (Fig. S5, B and C). CD8^+^ T cell percentages and numbers were also elevated within tumors injected with anti-LILRB4 antibody (Fig. S5, B and C). There was an increase in the percentage and number of CD4^+^ Teff cells and a decrease in Treg cell frequency, but not population (Fig. S5, B and C). CD8^+^ T cell and CD4^+^ Teff to Treg cell ratios are predictive of therapeutic efficacy of treatment in the B16 melanoma model (Quezada et al., 2006). We found a significant increase in both CD8^+^ T cell and CD4^+^ Teff cell to Treg cell ratios within tumor in anti-LILRB4–treated mice (Fig. S5 D). There was also an increase in CD8^+^GzB^+^/Treg cell and CD8^+^Ki67^+^/Treg cell ratios, suggesting anti-LILRB4 antibody treatment increases proliferation as well as granzyme B (GzB) production from CD8^+^ T cells (Fig. S5 D).

### LILRB4^−/−^ Treg cells and BMDMs are less suppressive than WT counterparts

**Figure 7.**
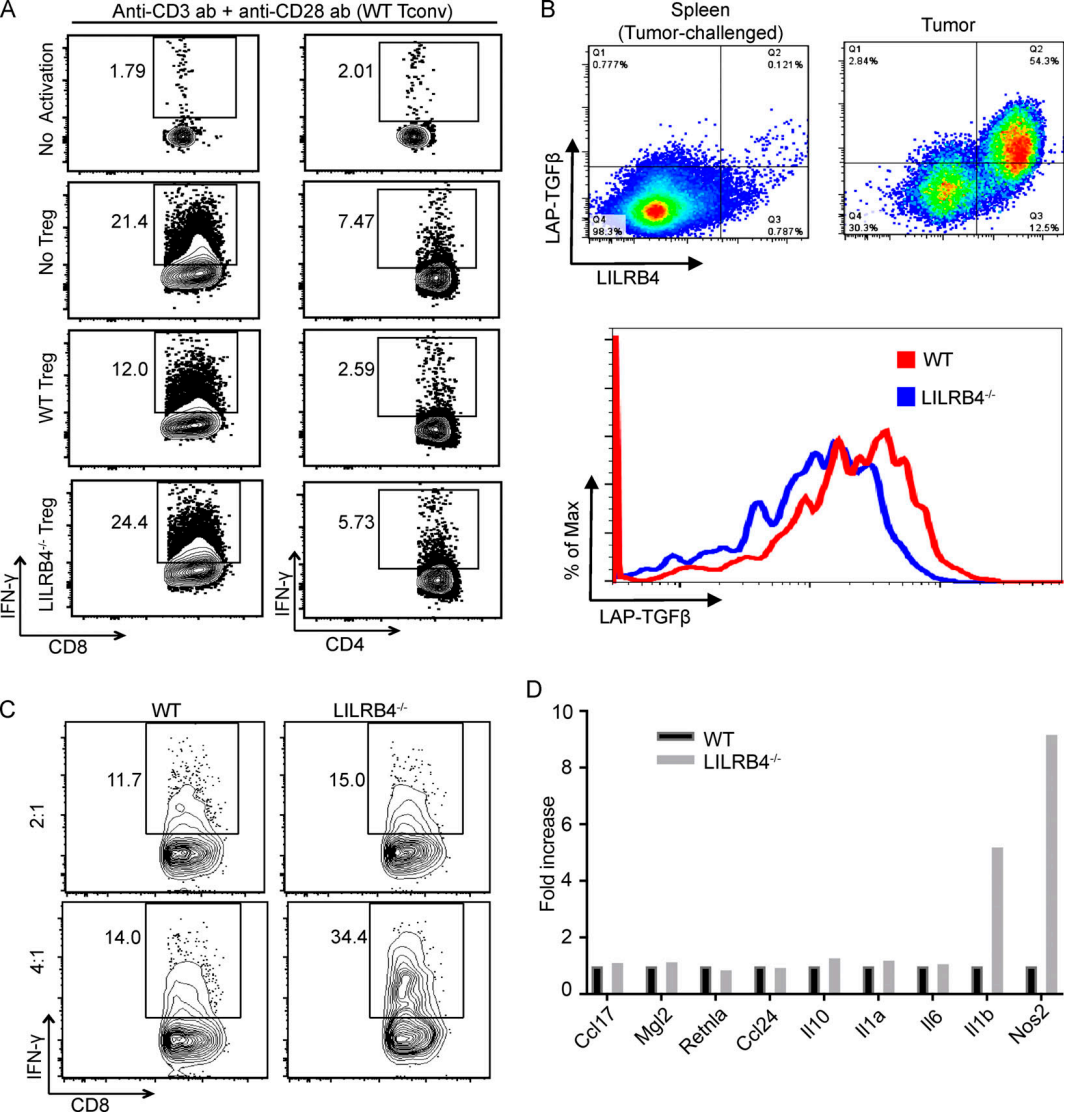
**Lack of LILRB4 reduces suppressive efficacies of Treg cells and bone marrow–derived M2-type macrophages.** **(A)** Treg cells and naive Tconv cells were isolated from spleens of LILRB4^−/−^ and WT mice as described in Materials and methods. LILRB4^−/−^ and WT Treg cells were then incubated with WT naive Tconv cells with simultaneous in vitro stimulation with anti-CD3 (1.25 µg/ml) and anti-CD28 (1.25 µg/ml) antibodies for 48 h. These cells were then stained with indicated antibodies. **(B)** Surface LAP-TGFβ expression is associated with LILRB4 expression. WT and LILRB4^−/−^ mice were challenged with 3 × 10^5^ B16/F10 cells intradermally on their right flanks. Tumors and/or spleens were dissected when the tumor grew to 1,000 mm^3^ and digested, and tumor-infiltrating cells and splenic cells were isolated and stained with indicated antibodies as described in Materials and methods and run on flow cytometer. Data are representative of two or three independent experiments with 5–10 mice per group. **(C)** BMDMs were generated from LILRB4^−/−^ and WT mice and were skewed to M2-phenotype as described in Materials and methods. These macrophages were then incubated in indicated ratio with naive untouched total T cells isolated from spleen with simultaneous in vitro stimulation with anti-CD3 (1.25 µg/ml) and anti-CD28 (1.25 µg/ml) antibodies for 48 h. Cells were then stained with indicated antibodies. **(D)** RNA was extracted from M2-skewed BMDMs, and RT-PCR analysis was performed as described in Materials and methods. Data are representative of two or three independent experiments with four to six mice in each group.

To analyze the suppressive activity of Treg cells, splenic naive T cells were isolated from WT and LILRB4^−/−^ mice using an affinity-based naive T cell isolation kit from Stem Cell Technology. These cells were stained with flow cytometry antibodies and FACS sorted as naive conventional T cells (Tconv cells; both CD8^+^ T cells and CD4^+^CD25^−^ T cells) and CD4^+^CD25^+^ Treg cells. WT conventional naive T cells were incubated with WT or LILRB4^−/−^ Treg cells and activated in vitro with anti-CD3 and anti-CD28 antibody. We observed less secretion of IFN-γ from CD8^+^ T cells and CD4^+^ T cells in the presence of WT Treg cells than LILRB4^−/−^ Treg cells, suggesting that LILRB4^−/−^ Treg cells were less suppressive than WT Treg cells (Fig. 7 A). This is an interesting observation, as inhibitory receptors may function differently on Treg cells as compared with Teff cells (Huang et al., 2004; Wing et al., 2008). TGFβ-induced suppression is one of the mechanisms by which Treg cells suppress Teff cell functions (Marie et al., 2005). Phenotypic analysis by flow cytometry of TILs from B16/F10-challenged tumor suggests that LILRB4^+^CD4^+^ T cells expressed higher surface LAP/TGFβ compared with LILRB4^−^CD4^+^ T cells. Therefore, we looked at the expression of surface LAP/TGFβ on WT and LILRB4^−/−^ Treg cells (Fig. 7 B). We found decreased surface LAP/TGFβ on LILRB4^−/−^ Treg cells compared with WT Treg cells, and this could be one of the mechanisms of reduced suppressive capability of LILRB4^−/−^ Treg cells compared with WT Treg cells.

We then analyzed the efficacy of LILRB4^−/−^ macrophages in suppressing T cell function. BMDMs from WT and LILRB4^−/−^ mice were skewed to M2-type phenotype in vitro by incubating them in the presence of IL-4. The conventional WT naive T cells were isolated from spleen by using an affinity-based naive T cell isolation kit; these cells were then activated with anti-CD3 and anti-CD28 antibodies in the presence of either WT or LILRB4^−/−^ M2-type macrophages for 48 h. These cells were analyzed for intracellular IFN-γ levels by flow cytometry. We found that WT conventional naive T cells (Tconv cells) secrete more IFN-γ in the presence of LILRB4^−/−^ macrophages compared with WT macrophages, suggesting that LILRB4^−/−^ bone marrow–derived (BMD) M2-type macrophages are less suppressive than WT BMD M2-type macrophages (Fig. 7 C). Our RT-PCR analysis of expression of cytokine genes in WT and LILRB4^−/−^ BMD M2-type macrophages suggest an increased expression of *IL1b *(IL-1β) and *Nos2* (iNOS2) cytokines transcripts in LILRB4^−/−^ M2-type macrophages compared with WT control M2-type macrophages (Fig. 7 D). These results suggest that lack of LILRB4 switches M2 macrophages toward a more inflammatory or M1-type phenotype.

### Anti-LILRB4 antibody reduces suppression by modulating intratumoral myeloid and T cells

**Figure 8.**
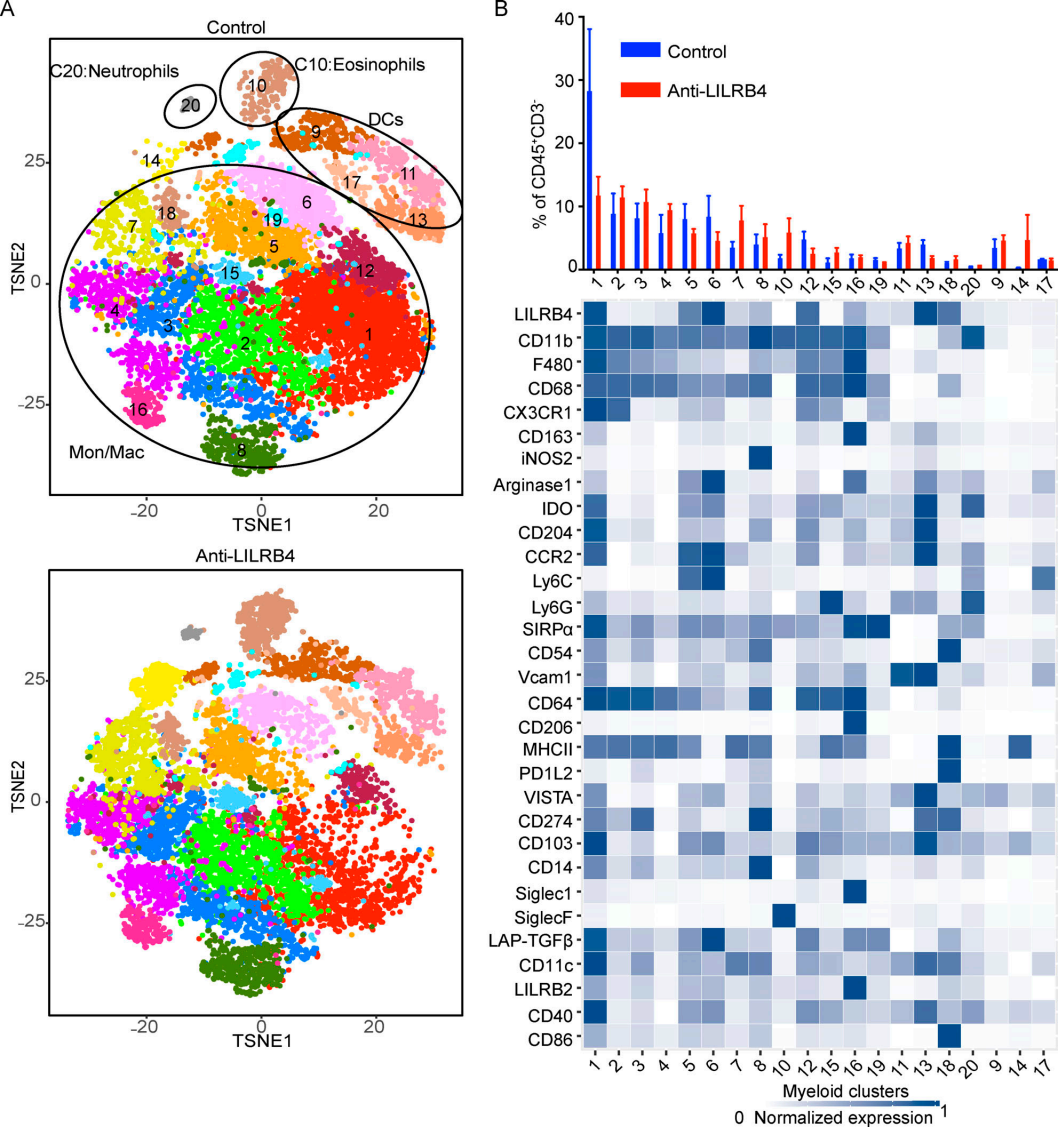
**Anti-LILRB4 antibody decreased the frequency of myeloid clusters associated with immunosuppressive phenotypes.** CyTOF proteomic analysis using a myeloid cell antibody panel for tumor-infiltrating cells from mice challenged with MC38 tumor cells and treated with anti-LILRB4 antibody or isotype control antibody. **(A)** t-SNE plot of an equal number of CD45^+^CD3^−^ MC38 tumor-infiltrating cells from each group and overlaid with color-coded clusters. Mon/Mac, monocyte/macrophage. **(B)** Bar plot of frequency of clusters and heatmap displaying normalized marker expression of each cluster. Cluster numbers are indicated on the x-axis. Data are representative of two independent experiments with four to seven mice per group.

To understand the mechanisms underlying the anti-LILRB4 antibody–mediated decrease in tumor growth and increase in survival, we analyzed the effects of anti-LILRB4 monoclonal antibody treatment on both intratumoral myeloid and lymphoid cell populations. To this end, mice were challenged with MC38 tumor and treated with anti-LILRB4 antibody on different days. Tumors were dissected, cells were isolated from tumors, stained with antibodies, and analyzed by CyTOF. Using the myeloid panel, 20 clusters were identified among CD45^+^CD3^−^ tumor-associated cells with relative frequency >0.5%. These clusters were annotated as 14 clusters of monocytes/macrophages (clusters 1–8, 12, and 14–16), four DCs clusters (clusters 9, 11, 13, and 17), one neutrophils cluster (cluster 20), and one eosinophils cluster (cluster 10; Fig. 8 A). Among monocyte/macrophage clusters, clusters 1, 5, 6, and 12 decreased in frequency after the treatment with anti-LILRB4 monoclonal antibody. Cluster 1 is CD11b^+^ F4/80^+^ CCR2^high^ CX_3_CR1^high^ Arg1^+^ IDO^high^ CD204^high^ VISTA^+^ LILRB4^high^ PD-L1^+^, cluster 5 is CD11b^+^ F4/80^low^CCR2^high^ Arg1^+^ CX_3_CR1^+^ IDO^+^ CD204^+^ LILRB4^+^ Ly6C^high^, cluster 6 is CD11b^+^ F4/80^low^ CCR2^high^ Arg1^high^ CX_3_CR1^+^ IDO^+^ CD204^+^ LILRB4^high^ Ly6C^high^, and cluster 12 is CD11b^+^ F4/80^+^ CCR2^+^ Arg1^+^ CX_3_CR1^+^ IDO^+^ CD204^+^ LILRB4^high^ (Fig. 8 B). The expression of Arg-1, CX_3_CR1, or IDO suggests that macrophage clusters that decrease in frequency with anti-LILRB4 have suppressive phenotypes (Biswas and Mantovani, 2010; Gubin et al., 2018; Murray et al., 2014; Ostuni et al., 2015; Qian and Pollard, 2010; Sica and Mantovani, 2012). Also, the clusters that were decreased in frequency after anti-LILRB4 antibody had high surface expression of CCR2 and CX_3_CR1, which are associated with poor prognosis in tumors (Lesokhin et al., 2012; Zheng et al., 2013; Grossman et al., 2018). Clusters 5 and 6 are identified as Ly6C^high^ CCR2^+^ circulating monocytes clusters, which are recruited to the tumor and become immunosuppressive TAMs (Franklin et al., 2014). The monocyte/macrophage clusters that were increased after the anti-LILRB4 monoclonal antibody include clusters 7, 8, 10, and 14. Cluster 7 is LILRB4^low^ CD11b^+^ F4/80^−^ CD68^+^ CX_3_CR1^−^ MHCII^+^ ICAM1^+^ IDO^−^ Arg1^−^ CD11c^+^, cluster 8 is LILRB4^low^ CD11b^high^ F4/80^+^ CD68^+^ iNOS2^+^ Arg1^−^ IDO^low^ PD1-L1^high^ CD14^high^ CD64^+^ CD11c^+^, and cluster 10 is CD11b^+^ F4/80^low^ Siglec-F^high^ eosinophils. Cluster 14 is CD11b^low^ CD11c^−^ CD54^+^CD103^+^ CCR2^+^ Ly6G^+^ MHCII^+^ VISTA^+^ LILRB4^low^ CD14^+^ CD40^+^ and expresses very low levels of CD11b in control, and this low CD11b expression goes away after anti-LILRB4 antibody treatment (data not shown). These clusters do not show phenotypes associated with suppressive macrophages, and therefore, interestingly, our data suggest a reduction in the frequencies of clusters with a suppressive phenotype and an increase in clusters with a nonsuppressive phenotype.

**Figure 9.**
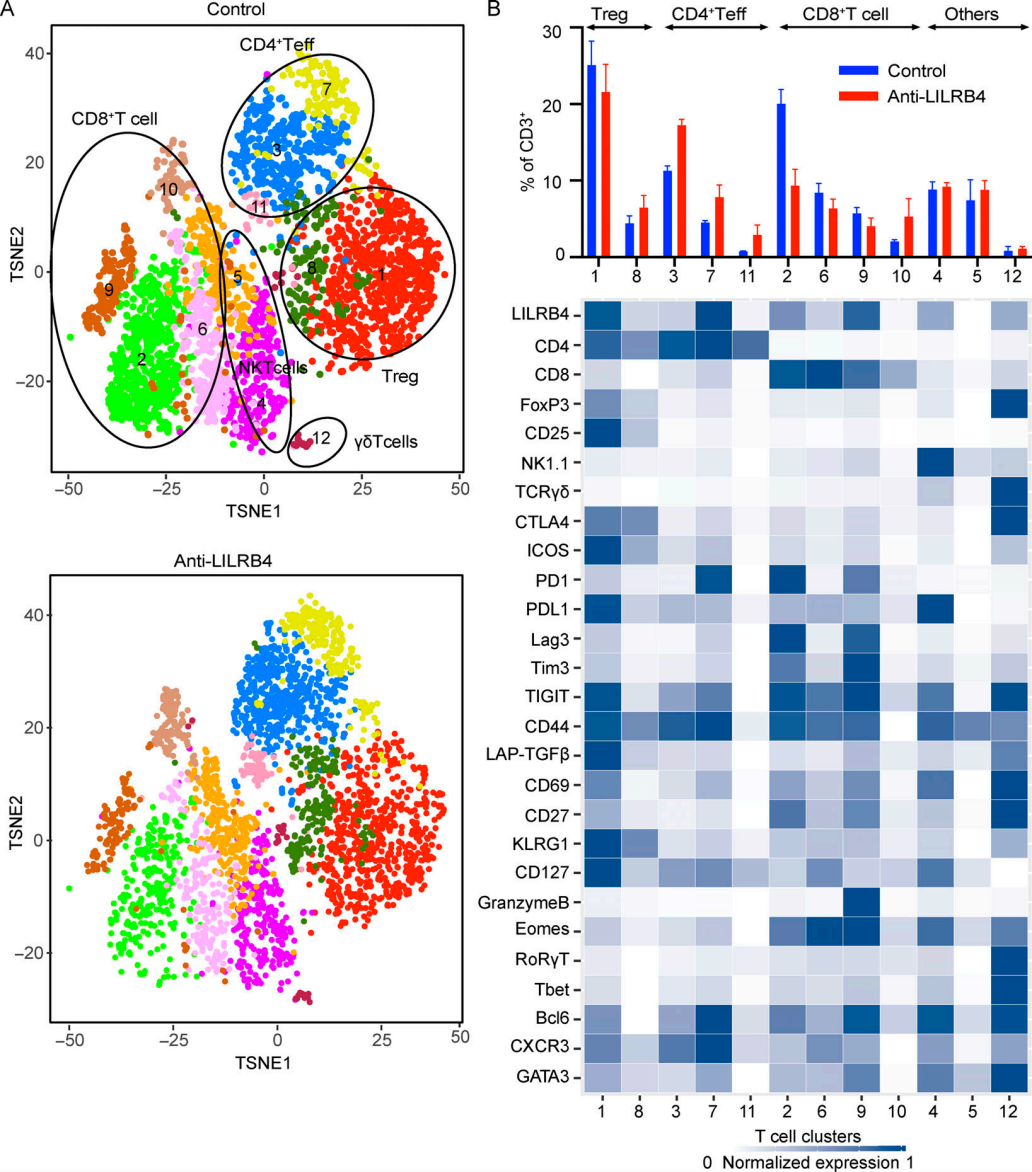
**CD4**^+^ **Teff cell clusters increased whereas exhausted CD8**^+^ **T cell clusters decreased in frequency after LILRB4 blockade.** CyTOF proteomic analysis using a T cell antibody panel for tumor-infiltrating cells from mice challenged with MC38 tumor cells and treated with anti-LILRB4 antibody or isotype control antibody. **(A)** t-SNE plot of an equal number of tumor-infiltrating CD3^+^ T cells from each group and overlaid with color-coded clusters. **(B)** Bar plot of frequency of T cell clusters and heatmap displaying normalized marker expression of each cluster. Cluster numbers are indicated on the x-axis. Data are representative of two independent experiments with four to seven mice per group.

As LILRB4 is expressed on T cells, we investigated the effects of anti-LILRB4 antibody on modulation of T cells by CyTOF. Twelve clusters were identified using T cell panels among tumor-infiltrating CD3^+^ T cells which were greater than 0.5% in relative frequency (Fig. 9 A). They were annotated as two clusters of Treg cells (cluster 1 and 8), three clusters of CD4^+^ Teff cells (clusters 3, 7, and 11), four clusters of CD8^+^ T cells (clusters 2, 6, 9, and 10), two clusters of NKT cells (clusters 4 and 5), and one γδ T cell cluster (cluster 12). Among Treg cell clusters, the LILRB4^high^ ICOS^high^ KLRG1^high^ LAP-TGFβ^high^ cluster decreased slightly in frequency, whereas the LILRB4^low^ ICOS^+^ KLRG1^+^ LAP-TGFβ^low^ cluster showed slight increase after anti-LILRB4 antibody treatment (Fig. 9 B). However, changes in Treg cell clusters were not significant. All CD4^+^ Teff cell clusters increased in frequency after anti-LILRB4 treatment, but only cluster 3 showed statistically significant increase. Cluster 3 comprises PD-1^−^ Tim-3^−^ LAG3^−^ CXCR3^+^ CD4^+^ T cells; lack of inhibitory receptors and expression of CXCR3 suggest this cluster comprises T helper type 1 (Th1) CD4^+^ T cells. However, expression of BCL6 also suggests that this cluster is in a transition state from Tfh-like cells to Th1 cells (Nakayamada et al., 2011). Among CD8^+^ T cell clusters, only cluster 2 showed statistically significant decrease in frequency after treatment with anti-LILRB4 antibody (Fig. 9 B). Cluster 2 is identified as a PD1^+^ LAG3^+^ Tim3^+^ exhausted CD8^+^ T cell cluster, and this cluster significantly decreased after anti-LILRB4 treatment. Cluster 9 is identified as a Tim3^+^ LAG3^+^ PD1^+^ exhausted CD8^+^ T cell cluster and showed slight non-significant decrease in frequency after LILRB4 treatment. Clusters 6 and 10 can be identified as the transitional “memory-like” or “precursor exhausted” T cells, as these clusters are PD1^−^ TIM-3^−^ LAG-3^−^ ICOS^+/−^ CD127^+^ CXCR3^+/−^ EOMES^+^ BCL6^+^ Tbet^+/−^ CD8^+^ T cells (Kallies et al., 2020). These CD8^+^ T cell subsets have been shown to be associated with checkpoint blockade efficacy in melanoma patients (Miller et al., 2019; Sade-Feldman et al., 2018). Cluster 6 does not change in frequency, but there was a slight non-significant increase in cluster 10. These results suggest that treatment of tumors with anti-LILRB4 antibody modulates CD4^+^ T cells toward a more Th1 effector phenotype. Among CD8^+^ T cells, there is a significant decrease in exhausted CD8^+^ T cells and an increase in memory-like CD8^+^ T cells. Taken together, these results reveal critical roles of LILRB4 in suppressing tumor immunity and its potential as a target for tumor immunotherapy.

## Discussion

We have identified myeloid inhibitory receptor LILRB4 as a potential target for immunotherapy, aiming to modulate the intratumoral macrophage–T cell relationship from pro-tumor to anti-tumor. We hypothesized that LILRB4 could play an important role in tumor immunity on the basis of our expression results, which showed LILRB4 expression in various solid tumors, including so-called cold tumors like pancreatic tumors, and high correlation with various inhibitory receptors. We explored the mechanisms of LILRB4 function using knockout mice and anti-LILRB4 antibody, and our findings suggest that LILRB4 inhibits tumor immunity through action on both myeloid and lymphoid compartments.

Our results, which showed reduced suppressive efficacy of LILRB4^−/−^ M2-type macrophages and increased T cell infiltration in LILRB4^−/−^ tumors, suggest that LILRB4 could be a potential target in cold tumors. We observed delayed tumor growth and prolonged survival in mT5 (murine pancreatic tumor model)–challenged LILRB4^−/−^ mice compared with WT mice.

Pancreatic cancer is lethal and resistant to traditional checkpoint blockade agents because of its immunosuppressive microenvironment, which consists of Treg cells and immunosuppressive M2-type TAMs (Le et al., 2013; Zhu et al., 2014; Knudsen et al., 2017; Sharma et al., 2017; DeNardo and Ruffell, 2019; Yang et al., 2021). Combining a checkpoint blockade antibody such as anti-CTLA4 or anti-PD1 with anti-LILRB4, which can have an impact on TAMs, could improve the clinical outcome of such patients. Our findings are consistent with studies by Deng et al., who showed that LILRB4 expressed on acute myeloid leukemia suppresses T cell activity (Deng et al., 2018). Similarly, studies by de Goeje et al. showed that LILRB4 is expressed on myeloid suppressor cells, and non–small cell lung cancer patients with a higher fraction of LILRB4^high^ subsets in myeloid cells had a shorter median survival compared with the patients with a lower fraction of LILRB4^high^ cells (de Goeje et al., 2015). In contrast to these studies, which showed that LILRB4 is expressed only on monocytic cells, we consistently found that LILRB4 is expressed on T cells as well as other cell types. These results are consistent with other studies, which showed expression of LILRB4 on other cell types, including NK cells, B cells, and T cells (Cella et al., 1997; Castells et al., 2001; Gu et al., 2003; Kasai et al., 2008; Fukao et al., 2014; Ulges et al., 2015). The discrepancies could be the result of differences in tumor types of cancer patients and use of different murine tumor models.

We tested a variety of murine solid tumor models for the role of LILRB4, which showed that LILRB4 is a potential target in solid tumors such as melanoma. We also showed the detailed expression of LILRB4 on various intratumoral cell types by flow cytometry, CyTOF, and scRNA-seq. We performed detailed mechanistic analysis of antibody efficacy by analyzing its effects on intratumoral myeloid and lymphoid compartments. Although we looked at the expression of LILRB4 in various cancer patients, our survival and mechanistic studies were restricted to mouse tumor models; these experiments should be repeated in humanized murine models. As we show LILRB4 is expressed on many cell types, understanding its functional mechanism in each cell type is also important. Despite its statistical significance, the survival advantage in mice treated with anti-LILRB4 is moderate; studies with other checkpoint inhibitors could help identify combinations that increase the survival rate more substantially. This is also quite in line with our findings that correlation between LILRB4 expression in tumors and survival in patients is observed but inconsistent among cancers. It is also important to note that there are some differences between human and murine LILRB4. Human LILRB4 has two extracellular Ig domains and three intracellular ITIM motifs, whereas murine LILRB4 (or gp49B) has two extracellular Ig domains and two intracellular ITIM motifs. Also, differences in their cellular expression patterns have been reported. Human LILRB4 has been shown to be expressed mostly on myeloid cells, whereas murine LILRB4 (or gp49B) has been shown to be expressed on both T cells and myeloid compartments. In spite of the limitation of our survival studies to murine tumor models, our analysis of the LILRB4 expression in tumors of cancer patients and TCGA data suggests that LILRB4 has an important role to play in cancer patients as well.

In conclusion, we have identified LILRB4 as a potential new target of tumor immunotherapy in solid tumors, effective either as monotherapy or potentially in combination with antibodies targeting T cells, and further studies could lead to development of novel immunotherapy drug for the treatment of cancer.

## Materials and methods

### Mice

6–8-wk-old C57BL/6J WT mice were purchased from Jackson Laboratory. LILRB4^−/−^ mice were obtained from Dr. Eric Long (National Institutes of Health, National Institute of Allergy and Infectious Diseases, Bethesda, MD). TRAMP^+^ mice were a gift from Dr. N. Greenberg (Prellis Biologics, Inc., Hayward, CA) maintained in hemizygous state. All mice were housed under specific pathogen–free conditions in accordance with institutional guidelines. MD Anderson Cancer Center’s Institutional Animal Care and Use Committee approved all animal experiments.

### Cell lines and reagents

The mouse melanoma cell line B16/F10 was obtained from Dr. Isaiah Fidler (MD Anderson Cancer Center [MDACC], Houston, TX) and maintained as described previously (van Elsas et al., 1999). The pancreatic cancer cell line mT5 was obtained from Dr. David Tuveson (Cold Spring Harbor Laboratory, Cold Spring Harbor, NY) and maintained as described previously (Boj et al., 2015). MC38 murine colon carcinoma cells were obtained from N. Restifo (National Cancer Institute, Bethesda, MD) and cultured in DMEM supplemented with 10% FBS with penicillin/streptomycin (P/S). The chemically induced murine bladder carcinoma MB49 cell line was kindly provided by Dr. A. Kamat (MDACC, Houston, TX) and cultured in DMEM with 10% FBS and P/S. The murine renal adenocarcinoma cell line and the RENCA cell line were obtained from the MDACC cell line core and maintained in DMEM with 10% FBS and P/S. The TRAMP-C2 cell line was derived from prostate tumor of a male TRAMP mouse and maintained as described previously (Foster et al., 1997). The following antibodies were used for flow cytometry analysis of tumors. Anti-mouse CD4 (clone GK1.5), anti-mouse CD8 (clone 53–6.7), anti-mouse CD45.2 (clone104), anti-mouse F4/80 (clone BM8), anti-mouse I-A/I-E (clone M5/114.15.2), anti- mouse TNFα, anti-mouse CD11c (N418), anti-mouse CD19 (6D5), anti-mouse NK1.1 (PK136), anti-mouse Tim-3, and anti-mouse CD160 (Clone 7H1) were purchased from BioLegend. Anti-mouse CD3 (clone 145-2C11), anti-mouse/human granzyme B (clone GB11), anti-human CD3, anti-human CD45, anti-human CTLA4, and anti-human PD-1 were purchased from BD Biosciences. Anti-mouse Foxp3 (clone FJK-16s), anti-mouse PD-1 (clone J43), anti-mouse CD272 (BTLA), anti-mouse 2B4 (244.2), anti-mouse PD-1H (VISTA), anti-mouse LAG-3, anti-mouse LILRB4, anti-mouse CD11b (clone M1/70), anti-mouse GR-1 (clone 1A8), anti-human CD279 (PD-1), anti-human CD223 (LAG-3), and anti-human LILRB4 (ILT3) were purchased from eBioscience. Functional anti-mouse CD3e monoclonal antibody (clone 500A2) and anti-mouse CD28 monoclonal antibody (37.51) were also purchased from eBioscience. Goat polyclonal anti-mouse LILRB4 (GP49B) antibody was purchased from Santa Cruz Biotechnology. Monoclonal anti-LILRB4 antibody hybridomas were generated in Armenian hamster. Hybridomas were selected, and supernatants from the resulting clones were screened by ELISA and their binding with the LILRB4-overexpressing cell lines by FACS. The selected clones were sent to BioXcell for large-scale purification of antibodies.

### Tumor challenge and treatment

Mice were given intradermal injections of 3 × 10^5^ B16/F10 cells or subcutaneous injections of 1 × 10^5^ mT5 cells, 3 × 10^5^ MC38 cells, 2 × 10^5^ MB49 cells, 2 × 10^5^ RENCA cells, and 4 × 10^5^ 4T1 cells on their right flanks on day 0. Mice were then treated with intraperitoneal injections of monoclonal anti–LILRB4 antibody (250 µg) or intratumoral injections of polyclonal anti–LILRB4 antibody (50 µg) on days 3, 6, 9, and 12. For rechallenged memory experiments, mice that survived primary tumor challenge and had been treated earlier with anti–LILRB4 antibody therapy were rechallenged with five times the above dose of tumor cells and left untreated. In experiments in which mice would be sacrificed on day 14 to understand the mechanism, the initial injection of B16/F10 cells was doubled to 6 × 10^5^ cells. These mice were sacrificed on day 14 to obtain tumors and draining lymph nodes or analyze tumor growth. For the tumor burden or survival experiments, the mice were considered moribund when the tumor grew to 1,000 mm^3^ and humanely killed.

### Tumor processing and flow cytometry

For phenotypic and functional analysis of tumor-infiltrating cells, mice were challenged and treated as described above. Mice from each treatment group were humanely killed on day 14, and their tumors and spleen were isolated. Isolated tumors were weighed, mechanically dissected, digested with DNase I and Liberase TL (Roche) at 37°C for 30 min, and then filtered through 70-µm nylon cell strainer. Spleens were mechanically dissected through a 70-µm nylon cell strainer and washed, and RBCs were lysed on ice for 5 min using RBC lysis buffer from Sigma-Aldrich. These cells were stained with Live/Dead fixable blue (Life Technologies) to exclude dead cells from analysis before staining with cell surface antibodies. These cells were further fixed and permeabilized with FoxP3 Fix/Perm buffer kit from eBioscience according to the manufacturer’s instructions and then stained with intracellular antibodies for further analysis by flow cytometry. Data were acquired on BD LSR II cytometer and analyzed by FlowJo Software.

### Human tumor analysis

Fresh tumors were manually minced before enzymatic digestion with 2 mg/ml collagenase A (Roche; catalog no. 11–088-793-001) and 40 U/ml DNase I (Sigma-Aldrich; catalog no. D5025) in DMEM and incubated with agitation at 37°C for 60 min. Following incubation, digests were passed through a 70-µm filter to remove residual particulates. Cells were then pelleted (centrifugation at 600 *g* for 5 min), washed in PBS, counted using a Trypan Blue exclusion viability dye, and repelleted before final resuspension at ∼1–5 million live cells/ml in cell culture freezing media comprised of 90% FBS and 10% DMSO (Sigma-Aldrich; catalog no. D2650). Samples immediately underwent controlled freezing (CoolCell LX) to −80°C before being moved into long-term liquid nitrogen storage. Cryopreserved tumor digests were thawed and mashed through 70-µm filters into RPMI-1640 with 10% FBS and P/S. Single-cell suspensions were then purified on a Histopaque-1119 (Sigma-Aldrich) discontinuous gradient centrifuged at 2,000 rpm for 20 min at room temperature. Live cells were then washed twice with FACS buffer and further used for RNA extraction for NanoString analysis or for flow cytometry analysis. These patients were treated at The University of Texas MDACC and had tumor samples collected and analyzed under Institutional Review Board–approved protocols (IRB 2012–0846; 2015–0041; PA13-0291; LAB00-063) and in accordance with the Declaration of Helsinki. All patients provided informed consent on The University of Texas MDACC institutional-approved laboratory protocol (PA13-0291) for tissue and blood collection for the study.

### RNA extraction from tumors and NanoString analysis

Mice were challenged and treated as described above and humanely killed on day 14 to isolate tumors. Isolated tumors were dissociated in the presence of Trizol reagent in gentleMACS M tubes by using gentleMACS dissociators. RNA was further extracted from dissociated tissue using a RiboPure RNA purification kit by following the kit manufacturer’s protocol. For each NanoString assay, total RNA from tumors was incubated overnight with NanoString code set mix at 65°C. The NanoString nCounter prep station was then loaded with this reaction mix cartridge for binding and washing. The cartridge was then transferred to the NanoString nCounter digital analyzer for scanning and data collection.

### Generation of BMD-derived M1/M2–type macrophages

For generation of BMDMs, WT and LILRB4^−/−^ mice were euthanized, and bone marrow was extracted from femurs and tibiae. Cells were cultured for 7 d in BMDM (RPMI 1640 containing Hepes plus L-glutamine plus 10% heat-inactivated FBS plus 1% P/S) supplemented with recombinant mouse macrophage colony-stimulating factor or granulocyte-macrophage colony-stimulating factor and incubated at 37°C, 5% CO_2_ concentration to generate BMDMs. After 7 d, these cells were activated for 1 d with LPS (1 μg/ml) plus IFN-γ (10 ng/ml) to convert them to M1 macrophages or with IL-4 (10 ng/ml) for conversion to M2 macrophages.

### Isolation of Treg cells

Naive spleens were isolated from LILRB4^−/−^ and WT mice. Single-cell suspensions were prepared from spleens, RBCs were lysed with RBC lysis buffer by incubating it on ice for 5 min, and splenocytes were filtered through cell strainers. Naive untouched total T cells were isolated by using negative T cell isolation kit (Stem Cell Technologies). These cells were then stained and sorted on BD FACSaria as naive conventional T cells (Tconv cells; both CD8^+^ T cells and CD4^+^CD25^−^ T cells) and CD4^+^CD25^+^ Treg cells.

### In vitro suppression assays

5–7 × 10^4^ WT conventional naive T cells were incubated with either WT or LILRB4^−/−^ Treg cells or BMDMs and stimulated in vitro in a 96-well round-bottom plate with anti-CD3 (1.25 µg/ml) and anti-CD28 (1.25 µg/ml) antibodies for 48 h. 0.5 µl/ml monensin (BD Biosciences) and 0.5 µl/ml brefeldin A (BD Biosciences) were added during the final 4 h of stimulation. Cells were then stained for surface, and these cells were further fixed and permeabilized with FoxP3 Fix/Perm buffer kit from eBioscience according to the manufacturer’s instructions and then stained with antibodies for intracellular proteins for further analysis by flow cytometry.

### Generation and screening of anti-LILRB4 monoclonal antibody

The monoclonal antibody generation was outsourced to the hybridoma center at Washington University, St. Louis, MO. For generating hybridomas, LILRB4 peptide-KLH was used to immunize Armenian hamster. Fusions were performed in Iscove DMEM supplemented with 20% FBS, 1mM sodium pyruvate, 4 mM L-glutamine, 50 U/ml penicillin, 50 μg/ml streptomycin, 50 μM 2-ME, and 1% Hybridoma Cloning Factor and selected by HAT (hypoxanthine-aminopterin-thymidine) medium. The primary method of screening of hybridoma supernatants was ELISA using peptide-BSA, and selected hybridomas were then screened with flow cytometry for binding to LILRB4-overexpressed cells. For flow screening, LILRB4-overexpressed cells and parental CHO cells were stained with LILRB4 antibody or culture supernatants at 4°C for 30 min. These cells were washed twice with FACS buffer and stained with anti–Armenian hamster secondary antibody conjugated with PE at 4°C for 30 min for further analysis by flow cytometry.

### Mass cytometry antibodies

Metal-conjugated antibodies were purchased from Fluidigm or unlabeled antibodies were purchased from various vendors and conjugated with metals in-house as per the manufacturer’s protocol (Fluidigm). Appropriate dilutions of each antibody were determined by serial dilutions of each antibody and staining relevant biological samples. The ideal dilution of each antibody was then found after staining analysis to minimize background and optimize detection of positively expressing populations.

### Mass cytometry analysis

Cryopreserved murine tumor digests (as described above) were thawed and mashed through 70-µm filters into RPMI-1640 with 10% FBS and P/S. Single-cell suspensions were then purified on a Histopaque-1119 (Sigma-Aldrich) discontinuous gradient centrifuged at 2,000 rpm for 20 min at room temperature. Live cells were then washed twice with FACS buffer and total concentration determined. 2.5 × 10^6^ cells obtained from tumors were then incubated with blocking buffer containing 2% of each bovine, murine, rat, hamster, and rabbit serum and 25 µg/ml 2.4G2 antibody at 4°C for 10 min before surface staining with antibody cocktail at 4°C for 30 min. Cells were then incubated with 195Pt cisplatin at 4°C for 1 min, washed twice with FACS buffer, and barcoded using palladium metal barcoding reagents according to the manufacturer’s protocol (Fluidigm). Cells were then fixed and permeabilized using the FoxP3 permeabilization kit (eBioscience) and stained with intracellular stain antibody cocktail for 30 min at room temperature. These stained cells were then washed twice with FoxP3 permeabilization buffer and twice with FACS buffer and incubated overnight in 1.6% PFA-PBS with Iridium nucleic acid intercalator. These cells were then washed with 0.5% BSA-PBS and filtered and then washed twice with 0.1% BSA water before analysis. Samples were then analyzed using Helios mass cytometer using the Helios6.5.358 acquisition software (Fluidigm). Mass cytometry data were normalized to EQ four element calibration beads signal using normalization software (Fluidigm), and mass tag barcodes were resolved using Debarcoder (Fluidigm). Samples were then manually gated for event length, live/dead discrimination, particular population, etc. in FlowJo. Data were then exported for downstream analysis and t-SNE (t-distributed stochastic neighbor embedding) dimension reduction, and Phonograph clustering analysis was done using the Cyt tool in MATLAB software.

### scRNA-seq

#### scRNA-seq library generation

Cryopreserved MC38 tumor digests (as described above) were thawed and mashed through 70-mm filters into RPMI-1640 with 10% FBS and P/S. Single-cell suspensions were then purified on a Histopaque-1119 (Sigma-Aldrich) discontinuous gradient centrifuged at 2,000 rpm for 20 min at room temperature. Live CD45^+^ cells were FACS sorted and encapsulated into droplets and libraries were prepared using Chromium Single Cell 30 Reagent Kits v3 according to the manufacturer’s protocol (10x Genomics). The generated scRNA-seq libraries were sequenced using an Illumina Novaseq 6000.

#### scRNA-seq data processing

Raw reads from scRNA-seq were aligned to the mm10 mouse reference genome and quantified using CellRanger count (v3.1.0). The individual count matrices were then merged using the CellRanger aggr pipeline. For detailed summary statistics, please see Table S1.

#### scRNA-seq data analysis with Seurat

The R package Seurat (v3.0.0) was used to analyze the merged dataset from two independent experiments. Briefly, genes expressed in less than three cells and cells that express less than 200 genes, more than 6,000 genes, or with percent mitochondria content larger than 10% were excluded from downstream analysis. S and G2/M cell cycle scores were assigned to each cell using the CellCycleScoring function with the predefined gene sets (Tirosh et al., 2016). Data were then normalized with a scale factor of 10,000 and scaled regressing out the latent variables Unique Molecular Identifiers, percent mitochondria content, S score, and G2/M score. Principal-component analysis was performed using the scaled data and the top 2,000 most variable genes. Clusters were identified using the first 50 principal components using a shared nearest neighbor modularity optimization-based clustering algorithm with a resolution of 0.6. Uniform manifold approximation and projection (UMAP) visualization used the same number of principle components and 30 neighbors (McInnes et al., 2018 *Preprint*). Only ptprc-positive clusters were used for generation of graphs.

### Statistical analysis

Data were analyzed with the GraphPad Prism 6.0 software program. The Student’s *t* test was used to assess differences between two groups for statistical significance. The Kaplan–Meier method was used to analyze survival data, and the log-rank (Mantel–Cox) test was used to assess differences in survival between different groups for statistical significance. P values < 0.05 were considered statistically significant.

### Datasets

All scRNA-seq datasets for MC38 tumor are available in the NCBI Sequence Read Archive BioProject database under accession no. PRJNA719748.

### Online supplemental materials

Fig. S1 shows that among CD45^+^ cells, LILRB4 is expressed largely on macrophages and Treg cells. Fig. S2 shows that LILRB4 is expressed largely on Treg cells, exhausted CD8^+^ T cells, and CD11b^+^ TAMs in the B16/F10 tumor model. Fig. S3 shows that LILRB4 expression is correlated with other inhibitory receptors and immune cell infiltrates. Fig. S4 shows that tumor-challenged LILRB4^−/−^ mice or WT mice given anti-LILRB4 treatment have reduced tumor burden compared with controls during primary challenge and rechallenge. Fig. S5 shows that anti-LILRB4 treatment decreased tumor weight and increased T cell frequencies, and Teff/Treg cell ratios in the TME. Table S1 shows a summary of statistics and quality control of alignment from CellRanger. Table S2 lists the top 25 genes differentially up-regulated in tumors of LILRB4^−/−^ mice compared with WT mice.

## Supplementary Material

Table S1 summarizes statistics and quality control of alignment from CellRanger.

Table S2 lists the top 25 genes differentially up-regulated in tumors of LILRB4^−/−^ mice compared to WT mice.

## Data Availability

Datasets are available upon request from the author. Materials generated in the course of this work, including the LILRB4 monoclonal antibody, may be obtained through a material transfer agreement.
