# Supplementary material for: LILRB4 suppresses immunity in solid tumors and is a potential target for immunotherapy
Source: J Exp Med. 2021 May 11;218(7):e20201811. doi: 10.1084/jem.20201811 (PMC8117208; doi:10.1084/jem.20201811)
Supplement: Table S1 — summarizes statistics and quality control of alignment from CellRanger. [file JEM_20201811_TableS1.docx]

**Table S1.** Summary of statistics and quality control of alignment from CellRanger.

| **Parameters** | **Batch 1** | **Batch 2** |
| --- | --- | --- |
| Estimated # of Cells | 10,567 | 7,980 |
| Fraction Reads in Cells | 91.80% | 92.50% |
| Mean Reads per Cell | 44,888 | 28,090 |
| Median Genes per Cell | 2,710 | 2,552 |
| Total Genes Detected | 18,341 | 16,801 |
| Median UMI Counts per Cell | 9,738 | 9,506 |
| Valid Barcodes | 97.60% | 97.00% |
| Reads Mapped Confidently to Transcriptome | 61.30% | 70.00% |
| Reads Mapped Confidently to Exonic Regions | 64.10% | 72.90% |
| Sequencing Saturation | 54% | 42.10% |
