# Supplementary material for: LILRB4 suppresses immunity in solid tumors and is a potential target for immunotherapy
Source: J Exp Med. 2021 May 11;218(7):e20201811. doi: 10.1084/jem.20201811 (PMC8117208; doi:10.1084/jem.20201811)
Supplement: Table S2 — lists the top 25 genes differentially up-regulated in tumors of LILRB4−/− mice compared to WT mice. [file JEM_20201811_TableS2.docx]

**Table S2.** Top 25 genes differentially upregulated in tumors of LILRB4^-/-^ mice compared to WT mice.

| **Gene name** | **Accession** | **Log2Fold Change** | **P-value** |
| --- | --- | --- | --- |
| Klra8 | NM_010650.3 | 3.67 | 0.00115 |
| Ccl19 | NM_011888.2 | 3.58 | 0.00409 |
| Pdcd1lg2 | NM_021396.2 | 3.53 | 0.00487 |
| Cd8a | NM_001081110.2 | 3.51 | 0.000196 |
| Il18rap | NM_010553.2 | 3.5 | 0.000343 |
| Tnfrsf11a | NM_009399.3 | 3.46 | 0.000911 |
| Cd79b | NM_008339.2 | 3.33 | 0.00111 |
| Klra6 | NM_008464.2 | 3.29 | 0.00123 |
| Il6 | NM_031168.1 | 3.18 | 0.00332 |
| C6 | NM_016704.2 | 3.16 | 0.00319 |
| Klrd1 | NM_010654.2 | 3.11 | 0.000482 |
| Il7 | NM_008371.2 | 3.1 | 0.000296 |
| Klrc1 | NM_001136068.1 | 3.04 | 0.00102 |
| Csf3r | NM_001252651.1 | 3 | 0.00103 |
| Btla | NM_177584.3 | 2.82 | 0.0032 |
| Icam4 | NM_023892.2 | 2.66 | 0.00871 |
| Abcb1a | NM_011076.1 | 2.62 | 0.00901 |
| Icos | NM_017480.1 | 2.59 | 0.00508 |
| Clec4e | NM_019948.2 | 2.58 | 0.00446 |
| Mx1 | NM_010846.1 | 2.53 | 0.00176 |
| Cd5 | NM_007650.3 | 2.48 | 0.00841 |
| Il27ra | NM_016671.3 | 2.43 | 0.00275 |
| Gzmb | NM_013542.2 | 2.43 | 0.00523 |
| Klrc2 | NM_001098669.1 | 2.4 | 0.0146 |
| Cd4 | NM_013488.2 | 2.39 | 0.0108 |
